# Status of Healthy Choices, Attitudes and Health Education of Children and Young People in Romania—A Literature Review

**DOI:** 10.3390/medicina60050725

**Published:** 2024-04-27

**Authors:** Alexandra-Ioana Roșioară, Bogdana Adriana Năsui, Nina Ciuciuc, Dana Manuela Sîrbu, Daniela Curșeu, Anca Lucia Pop, Codruța Alina Popescu, Monica Popa

**Affiliations:** 1 Department of Community Medicine, Iuliu Hațieganu University of Medicine and Pharmacy, 400349 Cluj-Napoca, Romania; alexandra.rosioara@umfcluj.ro (A.-I.R.); nina.ciuciuc@umfcluj.ro (N.C.); dsirbu@umfcluj.ro (D.M.S.); dcurseu@umfcluj.ro (D.C.); monica.popa@umfcluj.ro (M.P.); 2 Research Center in Preventive Medicine, Health Promotion and Sustainable Development, Iuliu Hațieganu University of Medicine and Pharmacy, 400349 Cluj-Napoca, Romania; 3 Faculty of Pharmacy, “Carol Davila” University of Medicine and Pharmacy, 020945 Bucharest, Romania; anca.pop@umfcd.ro; 4 Department of Abilities Human Sciences, Iuliu Hatieganu University of Medicine and Pharmacy, 400012 Cluj-Napoca, Romania; cpopescu@umfcluj.ro

**Keywords:** children, adolescents, nutrition, physical activity, health education, oral health, sexual education, mental health, alcohol consumption, tobacco use, social health, drug usage

## Abstract

*Background and Objectives*: This study aims to assess the health status and factors influencing healthy choices among children and young people in Romania, as well as the efficacy of related health education programs. Through understanding these dynamics, the study seeks to provide insights that can shape targeted interventions, policies, and educational strategies to improve this demographic’s overall health and well-being. *Materials and Methods*: For this study, we performed a literature review of original published papers on the health status, healthy habits, health education, predisposition to making healthy choices in the future, and accessibility to the paediatric health system of Romanian children and young people, as well as the effects of different types of educational interventions on this demographic in Romania. *Results*: The prevalence of dental caries is high in Romania. In terms of eating habits and nutritional status, a worrying proportion of children are overweight or obese, which can lead to a variety of future physical and psychological problems. In terms of physical activity, few adolescents demonstrate regular fitness practices. Romania presents an increase in alcohol and tobacco consumption among adolescents. The mental health of students has become a pressing public health concern, exacerbated by the COVID-19 pandemic. The use of social networks is linked to mental health issues among young people. Romania still has one of the highest rates of sexually transmitted diseases and faces a high incidence of cervical cancer, with a mortality rate three times higher than the EU average. High rates of teenage pregnancies are linked to limited information about sexuality and a lack of access to family planning at a young age. There are large discrepancies in the accessibility of medical services between urban and rural areas. *Conclusions*: Romania faces significant obstacles to providing high-quality healthcare to children and young people. Improving nutrition, immunisation rates, and access to medical services represent essential areas for enhancing the health of children and young people in Romania.

## 1. Introduction

The health status of children and young people should be one of the main concerns for decision-makers. Establishing healthy habits at these ages may guarantee a sustainable level of health for the following generations. Therefore, ensuring good health and well-being amongst school-aged children is a global public health priority. The contributions of schools to achieving this goal have increasingly been recognised [1]. Studying young people is crucial, as age strongly influences beliefs, knowledge, and behaviours. Adolescents and young adults tend to adapt rapidly to changes in their external conditions. Understanding who young people are today helps to predict their life trajectory and, consequently, the future of society [2].

According to the Centre for Disease Control and Prevention, self-reported health status is a subjective measure of an individual’s overall well-being. Importantly, this metric serves as a reliable predictor of significant health outcomes, including mortality, morbidity, and functional capacity [3].

The International Convention on the Rights of the Child defines a child as “any human being under the age of 18, except in cases where the law applicable to the child establishes the limit of majority under this age” [4]. For statistical purposes, the United Nations defines “youth” as anyone between the ages of 15 and 24 years old [5]. The European Commission defines young people as those between 15 and 29 years old [6]. European public health is monitored by both national agencies and NGOs [7].

During the 75th session of the UN Committee on the Rights of the Child from May to June 2017, the UN Committee drew Romania’s attention to the need for urgent action in important areas: (1) resource allocation, (2) discrimination against Roma children, (3) neglect, (4) sexual exploitation and abuse, (5) children deprived of a family environment, (6) children with disabilities, and (7) health services [8].

Since joining the EU, Romania has seen improved quality of life and healthcare initiatives, including those related to paediatrics and neonatal care. However, Romania continues to have the EU’s lowest health expenditure as a share of gross domestic product (GDP) and per inhabitant. Its healthcare system operates on three levels: primary, secondary, and tertiary [9]. Romania’s health objective is to promote healthy lives and well-being across all age groups. The 2030 Agenda established by the Council of Europe aims to reduce the prevalence of maternal and infant mortality, and the incidence of cervical and breast cancer, as well as reducing pregnancies in teenage girls, with a focus on disadvantaged and vulnerable groups [10].

As people advance in age, the choices they make affect their health and body even more. Healthy food choices, physical activity, and good sleep are essential for physical and mental well-being [11]. Unaddressed adolescent mental health conditions can persist into adulthood, potentially compromising physical and mental well-being and restricting prospects for a fulfilling life [12].

“Healthy choices” refers to the decisions, attitudes, and behaviours that promote and maintain physical, mental, and emotional well-being. These choices encompass various aspects of life, including diet, exercise, sleep, stress management, substance avoidance, and preventive healthcare practices.

Health education aims to (a) inform and educate the population about diseases and prevention, (b) promote healthy attitudes and skills, and (c) empower individuals to make decisions about their health [13]. In Romania, health education was introduced to schools in 2004 through the “Health Education in Romanian Schools” program, offering a flexible curriculum from grades I to XII [14]; however, only about 6% of students have access to this program [15]. In 2022, a law on healthcare reform was passed, which includes concrete data on how health education can be implemented in Romania such that it can be supported by specialist doctors, family doctors, resident doctors, nurses, midwives, community nurses, and representatives of institutions and organisations providing health education services [16].

As a part of research on the most important risk factors to the actual health status of the Romanian paediatric population and their proportion (% of all deaths), it was found that hypertension is the leading risk factor, followed by tobacco use, high cholesterol, and high BMI [17].

For children and young people, effective outpatient monitoring relies on close collaboration between family doctors, community paediatricians, and specialists in child and youth hygiene. If such collaboration fails, it can lead to the inadequate monitoring of children with chronic diseases, impacting both their health and the broader population’s well-being [18].

Regular prophylactic health check-ups play a vital role in assessing health at the individual and population levels, making them a cornerstone of public health and preventive medicine. Due to individual characteristics, children and young people are a crucial segment of the population, serving as indicators of community health and predictors of future generational well-being [19]. Romania requires a coherent, long-term strategy for paediatric healthcare to comprehensively address the existing challenges [20].

This study aims to map the currently existing data from the literature about the health status of children and adolescents from Romania to explore the factors influencing healthy attitudes, pointing out the sparsity of health education programs to underscore the need for further investigation.

Through understanding these dynamics, this study seeks to provide insights that can inform targeted interventions, policies, and educational strategies to improve this demographic’s overall health and well-being.

The importance of this study is crucial as, to the best of our knowledge, it is the first one of its type on the topic, bringing to light some of the gaps in the existing research on the subject.

## 2. Materials and Methods (Data Search)

For this study, we performed a literature review by searching for original research papers published on the health status of children and young people from Romania. This refers to the attitudes and lifestyle factors that influence the health status of children and young people, as well as the different types of educational interventions for helping young people adopt healthy habits in the future. The study also evaluated the outcomes of the oral health care system.

The questions used to conduct the literature review are as follows:

1. What is the current health status among children and adolescents in Romania, and what factors influence their healthy behaviours?
2. How do existing health education programs address these factors, and what gaps exist, highlighting the need for further research?

An exhaustive literature search was performed to locate all relevant studies. We searched for scientific articles published in the following major electronic databases: PubMed^®^/MEDLINE^®^ (National Library of Medicines, Bethesda, MD, USA), Web of Science^®^ (Clarivate Analytics, Philadelphia, PA, USA), and Scopus^®^ (Emeryville, CA, USA).

According to the study protocol, in PubMed, we used the database’s advanced search builder. We performed the search using MeSH terms with the main keywords “children and young people” initially, followed by AND/OR and “health education, health system Romania, COVID-19, alcohol use, tobacco use, teenage pregnancy, sexually transmitted diseases, nutrition, healthy habits, oral health, mental health, wellness, well-being”. Additionally, in Web of Science, we used the same keywords with the following filters applied: “randomised case-control, cross-sectional studies, interventional studies, on Humans, in the last ten years”.

We supplemented the search with a Google Scholar search for the relevant Romanian literature. There was no restriction on language, but only materials relating to a Romanian cohort (or subgroup) were included.

### Study Selection

Inclusion criteria: Articles published in the last 10 years (2013–2023) in the PubMed^®^ and Web of Science^®^ databases, in English language, and studies on children and young people, referring to the Romanian geographical area.

Exclusion criteria: Studies without a Romanian sample and those conducted on adults.

Data extraction: The following data were selected: author(s), year of publication, country, study aim, study design, and main results. Two reviewers (A.I.R. and B.A.N.), worked independently, extracted the data, and selected a sample of eligible studies, achieving good agreement. We calculated the kappa statistics to assess the inter-rater reliability; the kappa result was 0.8. First, the authors screened articles by title and abstract and then by full text. The studies were evaluated for eligibility according to the inclusion criteria. Our search strategy included snowball sampling of key papers. Duplicates and papers failing to meet the selection criteria were excluded.

**Figure 1:**
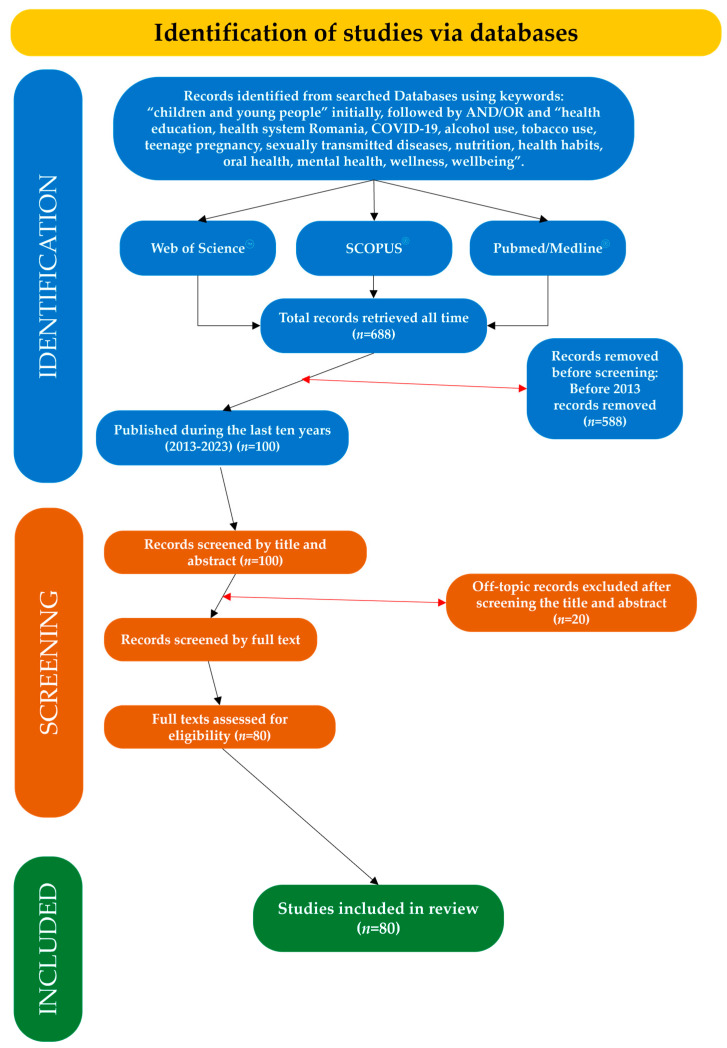
Reporting scheme for the present study, with a diagram illustrating the literature search process.

**Figure 2:**
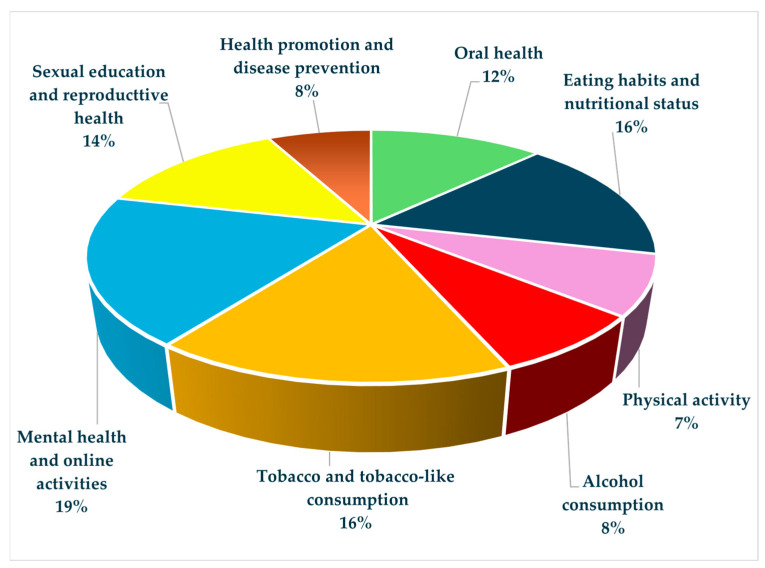
Graphical representation of the main topics approached in the literature published in the last 10 years on the following search topics: “children and young people” AND “health education, health system Romania, COVID-19, alcohol use, tobacco use, teenage pregnancy, sexually transmitted diseases, nutrition, healthy habits, oral health, mental health, wellness, well-being”.

This search strategy generated 688 articles. A time filter was applied, resulting in 445 articles, of which more than 100 were selected as relevant to the issue. From this selection, the full text was not available for 20 articles, leaving 80 studies to be reviewed. Due to the lack of data, some of the remaining articles were referenced as long as the abstract contained significant information. The focus was directly aimed at selecting the most recent data from the existing literature. Figure 1 illustrates the study selection flowchart and Figure 2 presents a chart of the studies selected in percentages for each main topic of the study.

## 3. Results

### 3.1. Oral Health

Oral health, which is critical throughout life, significantly impacts general health and social participation [21].

Our study analysed 10 papers that address oral health, 9 of which used questionnaires (standardised: 4; non-standardised: 5) regarding oral hygiene, knowledge, and attitudes. Only one of the studies used only a questionnaire as the method. Nine out of ten also performed clinical examinations and the results can be seen in Table 1. Two out of ten had interventions, where one had an intervention test group that received three experience lessons. Both the control group (who received a tooth brushing demonstration) and the intervention group showed improved oral hygiene and brushing frequency after the education sessions. The other interventional study performed oral health lessons, with a follow-up at two years, which showed good results; in particular, improved oral hygiene practices and an increase in the use of the correct tooth brushing technique were observed (Table 1). The extended data from the main findings of this section are detailed in Table S1 of the Supplementary Materials.

The prevalence of dental caries is still high in Romania. Romania’s dental care system operates primarily through private providers. However, a limited public program exists, partnering with select private practices to provide free emergency and preventive care for children under 18. These services are subject to a monthly budget restriction, potentially resulting in treatment delays [22]. Dental clinics are mainly located in urban areas, posing challenges to access in large rural regions. There are no national prevention or oral health education programs. There exists a paucity of data pertaining to the prevalence and incidence of dental caries, with existing studies focusing on small, locally selected samples. The lack of standardisation in diagnostic criteria makes it challenging to compare data. Notably, comprehensive national epidemiological surveys on oral health are yet to be conducted in Romania [22].

**Table 1:** Included studies that address oral health.

**Type of Study**	Cross-sectional (n = 7)							Interventional (n = 2)		Prospective (n = 1)
**Methods**	Questionnaire on oral health; clinical oral examination							Evaluation of oral health; oral health lessons; follow-up at two years		Questionnaire on oral health; clinical oral examination
**Populations**	879 aged 12–16 years, from three countries, Romania (n = 455)	814 aged 11–14 years	814 aged 11–14 years	718 aged 10–19 years	650 aged 10–19 years	258 aged between 16 and 69 years	98 aged 12 years	739 aged 11–16 years	76 aged 12–16 years	809 aged 6–8 years
**Reference**	[23]	[24]	[25]	[26]	[27]	[28]	[29]	[30]	[31]	[22]
**Category of issues treated**	**Main findings of all studies (n = 10)**									**Frequency (percentage)**
Oral health status	High prevalence of dental caries among adolescents (95.5% affected)									1 Study (9.09%)
	Average DMFT (number of decayed, missing, and filled teeth); similar in rural and urban areas; lower in females									1 Study (9.09%)
	High average DMFT and significant caries index									1 Study (9.09%)
Behaviours	Many adolescents do not brush in the evening or infrequently do so (33.7%)									1 Study (9.09%)
	A significant number skip regular dental check-ups (40.6%)									1 Study (9.09%)
	Tooth brushing duration varies, often not meeting recommendations									1 Study (9.09%)
Knowledge and attitudes	Most understand basic brushing; fewer know about remineralisation									1 Study (9.09%)
	Most perceive their oral health as moderate or good; females are more likely to report poor oral health									1 Study (9.09%)
Risk factors	Child DMFT is negatively associated with parental education									1 Study (9.09%)
	Age and consumption of sugary drinks are significant factors in tooth decay									1 Study (9.09%)
Interventions	Educational lessons improved tooth brushing technique									1 Study (9.09%)
	Educational intervention increased knowledge of fluoride benefits									1 Study (9.09%)

The colors represent the delimitation of the study: dark gray represents the first part of the table where I have type of study, methods, population and references displayed on vertical, and the light gray line referring to category of issues treated, main findings and percentage are for all studies listed up, listed on the horizontal.

Adolescents exhibit an alarmingly high caries prevalence (95.5%). This is worsened by poor oral hygiene (33.7% brush inconsistently in the evening and 40.6% lack annual check-ups) and the excessive consumption of sugar-sweetened beverages [27].

Educational attainment influences attitudes toward oral hygiene, with students often lacking a thorough understanding of recommended practices. Many students have limited knowledge about proper tooth brushing frequency, purpose, and timing. Implementing educational programs is crucial for improving students’ understanding and promoting positive attitudes toward oral hygiene [26].

Socio-economic factors, including parental education and living environment, along with the consumption of sugary foods and drinks, significantly influence both caries prevalence and access to dental treatment among Romanian schoolchildren [24].

Moreover, socio-economic variables play a role in the prevalence and severity of dental caries, leading to carious lesions and the lack of treatment among children aged 11–14 years in Romania [25].

Despite some positive oral health indicators (36.7% of 12-year-olds are caries-free and 38.8% brush twice daily), challenges remain in Romania, including frequent sugary food consumption, infrequent dental visits (11.2% of 12-year-olds never visiting a dentist), and a high mean number of decayed, missing, and permanently filled teeth (DMFT) (4.89) in 6-year-olds, exceeding the WHO recommendations [29,32]. Oral health and educational programs and experiential learning programs among 13–16-year-olds in Romania have demonstrated numerous positive effects on oral health knowledge and hygiene practices. These findings suggest the need for the widespread adoption of such interventions in schools [31].

In rural Romania, patients often rate their oral health as average to good. However, a study found that three-quarters have tartar deposits, and half have more than three cavities and at least one missing tooth. There is a significant gap between the reported oral health knowledge and actual practices, especially compared to urban areas. Despite satisfactory basic knowledge, rural patients’ practices do not match their perceived understanding [28].

In Romania, oral care services predominantly focus on treatment, with limited resources and attention allocated to preventive services [23]. Understanding oral health needs requires assessing public knowledge, perceptions, and habits. This information could guide policymakers in designing targeted education, implementing sustained prevention efforts, and ensuring everyone, regardless of location or gender, can access dental care [22,23].

### 3.2. Eating Habits and Nutritional Status

Healthy children study better. People with adequate nutrition are more productive and have a better chance of breaking the cycles of poverty and hunger [33].

In this section, we reviewed 13 studies that applied heterogenous methods, with seven applying questionnaires regarding eating habits, food behaviours, and eating lifestyle. Two of the studies were literature reviews regarding child obesity and its impact. Six of the studies conducted anthropometric measurements, one on skin examinations, one on consuming oral health, one on collecting data about blood pressure, and one on performing electro-cardiography and echo-cardiography exams. Only one of them used a standardised questionnaire. No intervention studies were registered in the selected studies (Table 2). The extended data from the main findings of this section are detailed in Table S2 of the Supplementary Materials.

Romania, along with other European countries, participates in COSI data collection, a tool developed by the World Health Organization’s Childhood Obesity Surveillance Initiative in Europe. This initiative gathers standardised data on children’s weight, lifestyle, and environment across countries to study the causes of obesity in the primary school context [47].

Romania faces a growing concern about children being overweight and obese, leading to health issues such as adult-onset hypertension and dyslipidaemia. The impact on children’s well-being and lifespan is unclear, but sustained community interventions aim to reduce these rates [42]. There exists a strong connection between childhood obesity and later cardiovascular problems, including early signs of heart disease and a higher risk of cardiovascular-related death in adulthood. The importance of and need for early obesity detection and prevention programs, along with obesity prevention and its complications, have increased. This demonstrates that obesity is much less understood than it first appeared [44]. Childhood obesity’s long-term effects necessitate immediate action to curb this trend. This requires collaboration between healthcare providers and all members of society to promote healthy living [46].

Despite progress in reducing the percentage of underweight children, Romania faces a challenge, with almost a quarter of its children being overweight or obese (according to the WHO criteria) [48]. Studies in Romania have identified male gender, pre-puberty, and urban living as risk factors for children being overweight [43]. Obesity is most prevalent in children with high fast-food intake and low physical activity. Parents’ weight perception of their children may also influence weight gain [39].

A Bucharest study revealed high obesity rates in children and adolescents aged 6–18 years. This was worse for younger children and boys. Interestingly, unhealthy eating habits were common, despite no clear connection to weight status [36]. A northwest Romanian study found similar overweight/obesity rates across the same standards (~27–29%). Overweightness peaked in prepubescent children (up to 10–12 years) and declined in older adolescents (17–18 years), regardless of the classification method used [34].

A cross-sectional study found that age, BMI, and diet influenced periodontal bacteria and salivary pH in children and adolescents (particularly within the first 6 months of life) [41].

Boys in Romania face higher rates of overweightness or obesity than girls. The shift from communism brought unhealthy dietary habits, including the excessive consumption of fast food, sugary drinks, and sweets. Sedentary lifestyles, influenced by exposure to electronic devices and screens, have contributed to the rise in paediatric obesity [45].

Misperceptions about children’s actual weight by both children and parents potentially impact their current lifestyle and delay intervention in weight management. Factors contributing to misperceptions include body mass index, gender, weight-related behaviours, parents’ estimations, family discussions, bullying, online bullying, and exclusion from groups [35]. There is a need for education for both parents and children to correctly identify and manage children’s body weight [38].

During COVID-19, students with high screen time (4–6+ h) were more likely to have unhealthy diets (high comfort foods and low vegetables). BMI was linked to both parental BMI and screen time. Reducing screen time and improving dietary habits could help address rising student obesity rates [40].

Adolescents display diverse eating patterns, reflecting economic changes marked by decreased fruit and vegetable consumption and increased empty-calorie foods. This trend contributes to a rising generational cardiovascular risk. Medically supervised low-carb diets starting at age 14 have proven effective in addressing metabolic syndrome components, offering a potential solution for reducing cardiovascular risk. Adolescents on this diet prefer carbohydrates to fibre-rich vegetables, low-glycaemic fruits, and small amounts of whole grains, pasta, rice, potatoes, lentils, or corn [49].

Statistically significant associations link age to family-set mealtime rules compliance and receiving an eating behaviour assessment. Education level correlates with knowledge of the health benefits of vegetable oils and an understanding of the essential role of dietary behaviour in disease prevention [50].

In Romania, recent attention to nutrition, driven by the alarming rise in obesity rates, emphasises healthy eating behaviours, which are crucial in a child’s upbringing and are influenced by economic possibilities and the family’s level of education [37].

### 3.3. Physical Activity

Exercise of any intensity improves health [51].

In this section, we reviewed six papers; five of them applied questionnaires (one non-standardised and four standardised) but each of them used different standards. One of them performed a clinical examination on oral hygiene and only one of the studies used intervention, consisting of video exercises for physical activity during school classes. This interventional study confirmed the positive impact that video exercises had on learning (Table 3). The extended data from the main findings of this section are detailed in Table S3 of the Supplementary Materials.

Lack of physical activity, sedentary behaviour, and sleep significantly impact children’s health. A cross-sectional study on socio-economic disparities in these factors across 24 countries in the WHO European Region revealed diverse patterns. High socio-economic status (SES) children use more motorised transport, while low SES children join fewer sports clubs. SES does not clearly impact active play, but it is linked to sleep variation. Overall, low SES consistently correlates with unhealthy behaviours, demanding targeted public health solutions [52]. Unlike adult athletes, competitive sports seem to have a positive impact on children’s oral hygiene, periodontal health, and dental integrity, according to one study [53].

Applying the Youth Physical Activity Promotion (YPAP) framework showed that ability and self-worth strongly influence physical activity in Romanian students, alongside other predisposing, enabling, and reinforcing factors [54].

A study found that young adults’ physical activity levels strongly influence heart rhythm patterns (FQRST on ECG). Higher activity correlated with a narrower FQRST, while sedentary behaviour led to wider angles. Furthermore, researchers found a link between BMI and FQRST [55].

A study revealed high physical activity in adolescent boys, nutrition awareness in girls, and a strong link between self-perceived body attractiveness and weight status. It also highlighted the need for school-based nutrition education [56]. Another study found that pandemic-induced telework led to increased screen time, decreased physical activity, weight gain, and poor sleep quality (less than 6 h on some weeknights) among young computer workers [57].

**Table 3:** Included studies that address physical activity.

**Type of study**	Cross-sectional study (n = 4)				Interventional (n = 1)	Randomised control trial (n = 1)
**Methods**	Questionnaire; anthropometric measurements; interview (face-to-face); clinical measurements of ECG				Questionnaire based on the Attitudes toward Physical Activity Scale (APAS); intervention: exercise videos provided by HOPSports Brain Breaks^®^	Clinical examination: OHI-S (Oral Hygiene Index-Simplified); PMA (Papillary–Marginal–Attached Gingival Index) and DMF-T values were determined
**Populations**	124,700 aged 6–9 years from 24 countries, including Romania (n = 9094)	1320 aged 14–18 years	665 aged 18–23 years	124 aged 18–27 years	3036 aged 8–11 years from 8 countries, including Romania	173 aged 6–17 years
**Reference**	[52]	[56]	[54]	[55]	[58]	[53]
**Category of issues treated**	**Main findings of all studies (n = 6)**					**Frequency (percentage)**
SES (socio-economic status) and activity	Higher SES linked to increased use of active transportation (home to school)					1 Study (14.2%)
	Lower SES linked to lower sports participation and more screen time (>2 h/day)					1 Study (14.2%)
	Lower parental education is associated with less time spent in sports activities					1 Study (14.2%)
Sports and health	Competitive sports linked to better oral hygiene (compared to the control group)					1 Study (14.2%)
	Lack of studies on the impact of competitive sports on children’s oral health identified					1 Study (14.2%)
Physical activity and mental health	All the theoretical dimensions of the YPAP model (predisposing, enabling, and reinforcing) positively influence PA					1 Study (14.2%)
Health	Wider FQRST complex (electrical heart activity) linked to lower physical activity levels (PALs)					1 Study (14.2%)

The colors represent the delimitation of the study: dark gray represents the first part of the table where I have type of study, methods, population and references displayed on vertical, and the light gray line referring to category of issues treated, main findings and percentage are for all studies listed up, listed on the horizontal.

### 3.4. Alcohol Consumption

In this section, we examine six studies which all applied questionnaires, with only one of these being standardised. All of the examined studies indicated an increase in alcohol consumption in young people, starting at 15 years of age. These studies place Romania above the average of European countries. There were no interventional studies found during the research (Table 4). The extended data from the main findings of this section are detailed in Table S4 of the Supplementary Materials.

Younger people are significantly impacted by alcohol, with a staggering 13.5% of deaths in the 20–39 age group attributed to alcohol. Disadvantaged groups face higher rates of alcohol-related death and hospitalisation. In Romania, per capita alcohol consumption exceeds European averages for boys in the 15 to 19 and 20 to 24 age groups. Girls in the 20 to 24 age category also surpass the European average. The European School Survey Project on Alcohol and Other Drugs (ESPAD) 2019 in Romania shows an increase in alcohol consumption among adolescents, particularly in boys (82.1% lifetime alcohol consumption), compared to the European average (78.5%) [59,60,61].

Effective intervention strategies are vital to mitigate student alcohol consumption and its ramifications. Studies on medical students reveal significant risky behaviour, with males showing higher drug use and physical activity levels. Both male and female drinkers engage in risky behaviours. Additionally, a high prevalence of alcohol use among students—especially males—is noted, with poor eating habits linked to low fruit and vegetable consumption. Health promotion campaigns targeting alcohol, smoking, nutrition, and drug use are recommended for improved student performance [62,63,64].

When examining choices, the primary motivations for alcohol consumption include taste, sensory experience, relaxation, and social interaction. Understanding students’ drinking behaviour and taste preferences is essential for creating strategies to discourage excessive alcohol consumption. Craft beer, considered a healthier and safer alternative, could play a role in reducing high-proof alcohol consumption [65].

**Table 4:** Included studies that address alcohol consumption.

**Type of study**	Cross-sectional (n = 6)					
**Methods**	Questionnaire on alcohol consumption and lifestyle					
**Populations**	99,647 aged 15–16 years from 35 countries, including Romania (n = 3764)	1727 aged 17 years	1212 aged 18–25 years	1044 aged 18–24 years	722 aged 18–25 years	468 aged 18–25 years
**Reference**	[60]	[66]	[64]	[65]	[63]	[62]
**Category of issues treated**	**Main findings of all studies (n = 6)**					**Frequency (percentage)**
Prevalence	Alcohol consumption prevalence among students: 79.9–82.1%					3 Studies (18.75%)
	Abstinence prevalence: males (10.8%) lower than females (17.6%)					1 Study (6.25%)
	Binge drinking prevalence: males (18%)					1 Study (6.25%)
Gender	Males drink more than females per week					1 Study (6.25%)
	Males have higher prevalence of binge drinking					1 Study (6.25%)
	More risky behaviour (smoking and drugs) was observed in females who drink more compared to low-risk females					1 Study (6.25%)
	Females prefer sweet alcoholic beverages (speciality beers)					1 Study (6.25%)
Correlations	Positive association between drinking and gender, physical activity, smoking, and fast-food intake					1 Study (6.25%)
	Negative association between drinking and sleep duration and study hours					1 Study (6.25%)
	Alcohol use correlates with low-prevalence illicit substance use (1.6%)					1 Study (6.25%)
Age of onset	Statistically significant correlation between age of initial alcohol consumption and family alcoholism					1 Study (6.25%)
Motivations	Primary motivations for drinking include taste, relaxation, social interaction, and sensory experience					1 Study (6.25%)
Locations	Occasional drinking in public places is preferred by both males and females					1 Study (6.25%)
Types of drinks	Beer is most common (males: 81.3%; females: 66.5%), followed by spirits (males: 40.8%; females: 28.6%)					1 Study (6.25%)
Drinking	Most frequent drinking situations: parties (62.7%); with peers (37.5%); clubs (25.2%)					1 Study (6.25%)
Comparison	Alcohol consumption in Romania is higher than European average (78.5%)					3 Studies (18.75%)

The colors represent the delimitation of the study: dark gray represents the first part of the table where I have type of study, methods, population and references displayed on vertical, and the light gray line referring to category of issues treated, main findings and percentage are for all studies listed up, listed on the horizontal.

### 3.5. Use of Tobacco and Tobacco-Like Products

In this section, we review 13 papers. One of the studies was an interview study and 12 of the studies used questionnaires: two of them were standardised and one performed anthropometric measures. Three of the studies were interventional studies: one of them used an intervention with video materials about the effects of smoking and group discussions. Another interventional study included five lessons on smoking prevention. The third interventional study had smoking prevention lessons that used the ASPIRA (a web-based, multimedia smoking prevention program for adolescents in Romania) standardised program (adapted from the ASPIRE Program), which offers diverse learning experiences using animations, videos, quizzes, and games, structured into five learning modules (Table 5). The extended data from the main findings of this section are detailed in Table S5 of the Supplementary Materials.

In central and eastern European countries, young people’s susceptibility to tobacco use is influenced by social, educational, and political factors, as well as attitudes toward tobacco. Gender, disposable income, exposure to ads, and perceptions of smokers’ social status are key influences. These factors should shape anti-tobacco efforts aimed at youth [67].

In Romania, adolescent tobacco consumption remains high (lifetime consumption: 49.5%), with similar rates for boys and girls. Smoking experimentation is linked to smoking knowledge, academic performance, exposure to tobacco smoke at home, and smoking among peers. Notably, those reporting a significant proportion of smokers among peers are nine times more likely to smoke [61,68].

A Romanian study found links between e-cigarette use, smoking, alcohol, and illicit drug use, and the intention for future e-cigarette use [69]. Another study highlights that time spent using the internet and smoking correlates with palpitations among students aged 13 to 15 [70].

In rural areas of Romania, 96.3% of middle school students are aware of e-cigarettes, with experimentation rates higher among smokers. Factors influencing experimentation include associating with peers who have tried e-cigarettes and assuming that this will lead to stopping the habit. The decision to use heated tobacco products is influenced by legislative gaps, product attractiveness, and perceived less harmful effects [71,72].

**Table 5:** Included studies that address the use of tobacco and tobacco-like products.

**Type of study**	Cross-sectional (n = 10)										Prospective (n = 2)		Interventional (n = 1)
**Methods**	Questionnaire on smoking habits and lifestyle; interview; clinical measurements										Questionnaire; smoking prevention lessons		Questionnaire on tobacco use behaviours; intervention with video materials about effects of smoking and group discussion
**Population**	99,647 aged 15–16 years from 35 countries, including Romania (n = 3764)	12,328 aged 13–17 from 11 countries (Romania: n = 1140)	10,783 aged 11–17 years, from five countries, including Romania (n = 3718)	1313 aged 12–15 years	1147 aged 13–15 years	783 aged 13–14 years	675 aged 14–15 years	400 aged 18–24 years	275 aged 10–18 years	30 aged 18–26 years	1369 aged 14–15 years	1071 aged 14–15 years	275 aged 14 years
**Reference**	[60]	[73]	[67]	[68]	[70]	[71]	[74]	[69]	[75]	[72]	[76]	[77]	[78]
**Category of issues treated**	**Main findings of all studies (n = 13)**												**Frequency (percentage)**
Prevalence	≈25% of adolescents show susceptibility to tobacco use across European countries												1 Study (10%)
	Lifetime tobacco use among Romanian adolescents has a downward trend, currently at ≈50%												1 Study (10%)
	E-cigarette awareness is high among middle school students (96.3%)												1 Study (10%)
Attitudes and beliefs	Beliefs about smoking can influence initiation (e.g., fewer friends; less attractive)												1 Study (10%)
	Certain beliefs make adolescents MORE susceptible to smoking (e.g., more friends: Romania)												1 Study (10%)
Correlates and risk factors	Classmate smoking behaviour is a major predictor of adolescent smoking												1 Study (10%)
	Disposable income is positively associated with smoking susceptibility (most countries)												1 Study (10%)
	Urban residence, internet use, and energy drink consumption linked to palpitations (correlated with substance use)												1 Study (10%)
Interventions	Anti-smoking education programs can reduce the risk of smoking initiation in Romania												1 Study (10%)
	Program effectiveness may be influenced by individual factors (father’s education, etc.)												1 Study (10%)

The colors represent the delimitation of the study: dark gray represents the first part of the table where I have type of study, methods, population and references displayed on vertical, and the light gray line referring to category of issues treated, main findings and percentage are for all studies listed up, listed on the horizontal.

Regarding the likelihood of becoming a smoker in child and youth care systems, the probability varies, with children raised in professional caregiver environments more prone to early tobacco initiation. Prevention programs, particularly in schools, prove effective in reducing smoking initiation among Romanian adolescents [75,77,78].

The ASPIRA program, adapted from the US-developed ASPIRE program, shows promise in reducing smoking initiation among adolescents in central and eastern Europe. It identifies behavioural risk factors and addresses socio-demographic, psychological, and behavioural factors to enhance effectiveness [74,76].

Smoking remains a significant public health issue among Romanian adolescents, with links to mental health problems and family disorganisation. Early preventive measures are essential for both adolescents and their parents [73].

### 3.6. Mental Health and Well-Being (Both before and after the COVID-19 Pandemic) and Online Activities

Mental health is not just the absence of disorders; it is a complex, individual experience with varying degrees of challenge and a wide range of social and clinical impacts [79].

In this section, we examine 15 papers. All of them were questionnaire-based, with seven being standardised, but each used different standards. Eight of the questionnaires used non-standardised questionnaires. The questionnaires focus on many different areas, investigating stress anxiety, depression leading to missing daily interaction, body image, self-esteem, levels of loneliness, social network addiction, family satisfaction, subjective happiness, perfectionism, behavioural problems, perception of the pandemics, coping mechanisms, online learning, parenting styles, school burnout, emotion regulation, level of security, social support, school social climate, and attitudes regarding digital health training. One of them also used an interview method on children. We did not find any interventional study during our research (Table 6). The extended data from the main findings of this section are detailed in Table S6 of the Supplementary Materials.

During adolescence, individuals facing frequent and intense negative events often exhibit higher levels of depression and anxiety symptoms [80]. The pandemic, while introducing new stressors, has indirectly spurred a heightened focus on the mental health of students, necessitating specialised interventions for medical students dealing with multi-faceted challenges such as gender and financial issues [81].

Students’ mental health has become a pressing public health concern, exacerbated by the challenges of the pandemic. Remote learning disrupts routines that are vital for student mental health, potentially increasing dropout rates. Authorities and universities must prioritise solutions to prevent long-term damage to students’ well-being and academic success [82].

The use of social networking sites (SNSs) seems to be related to mental health issues among young people.

Snapchat boosted self-esteem, whereas TikTok harmed weight perception, yet participants showed high body esteem along with significant loneliness (especially in younger users) and males reported better mental health than females [83]. For individuals aged 13–35, Facebook engagement is negatively linked to family satisfaction and positively associated with depression symptoms. Higher Facebook intensity correlates with increased Facebook addiction [84]. Perfectionism, especially self-critical and narcissistic forms, is linked with internet addiction in students. This can lead to health-risk behaviours, subsequently harming their happiness [85].

Compared to other European countries, Romania has the lowest rate of internalisation problems (2.4%, contrasting with 11.1% in Iceland), with boys more likely to report such problems than girls. In nations with unequal income distribution, adolescents tend to have lower life satisfaction, especially those making unfavourable self-judgments in non-equal environments [86].

**Table 6:** Included studies that address the mental health and well-being situation (both before and after the COVID-19 pandemic) and online activities.

**Type of study**	Cross-sectional (n = 15)														
**Methods**	Questionnaire on stress, happiness, substance use, the pandemic, online education, mental health, image of their own body, self-esteem, school burnout, anxiety, and depression														
**Populations**	8952 aged 14–17 years from 5 countries; Romania (n = 1830)	2690 aged 15–16 years	1061 aged 18–24 years	993 aged 13–16 years from 6 countries; Romania (n = 190)	722 aged 18–24 years	708 aged 13–35 years	604 aged 18–22 years	602 aged 8–16 years	461 aged 18–25 years	427 aged 18–24 years	383 aged 18–22 years	306 aged 22–25 years	155 aged 10–13 years	85 aged 16–19 years	73 aged 5–18 years and 73 parents aged 29–47 years
**Reference**	[86]	[80]	[81]	[87]	[82]	[84]	[88]	[89]	[85]	[83]	[90]	[91]	[92]	[93]	[94]
**Category of issues treated**	**Main findings of all studies (n = 15)**														**Frequency (percentage)**
Prevalence and symptoms	High prevalence of depression and anxiety symptoms in adolescents														2 Studies (15.38%)
	Recent, intense negative life experiences are linked to increased depression symptoms (35.8% of adolescents)														1 Study (7.69%)
	Depression scores decreased from 2018 to 2022														1 Study (7.69%)
Risk factors	Exposure to adversity is linked to increased mental health problems														1 Study (7.69%)
	Academic stress, financial difficulty, being female, and pre-clinical years increase depression risk														1 Study (7.69%)
	Losing a family member to COVID-19 is linked to increased depression and anxiety														1 Study (7.69%)
	Boredom, missing social interaction, phone overuse, and pandemic news interest are linked to higher stress														1 Study (7.69%)
Social media and technology	Negative comparisons on social media are common (≈50% of students)														1 Study (7.69%)
	Snapchat use is linked to higher self-esteem; TikTok use is linked to lower weight status														1 Study (7.69%)
	Facebook use is linked to decreased family satisfaction and increased depression														1 Study (7.69%)
	Facebook overuse is linked to addictive behaviours														1 Study (7.69%)
COVID-19 impact	Children associate masks with COVID-19; sadness is the most common feeling														1 Study (7.69%)
	Moderate pandemic-related hardship, mainly due to online classes														1 Study (7.69%)

The colors represent the delimitation of the study: dark gray represents the first part of the table where I have type of study, methods, population and references displayed on vertical, and the light gray line referring to category of issues treated, main findings and percentage are for all studies listed up, listed on the horizontal.

Concerning the pandemic’s perceived adverse effects, there is a moderate level, primarily due to the shift to online schooling. Children’s self-reported harm is linked to perceived adverse effects on their families. Dissatisfaction with online classes is widespread, but positive thoughts and family relationships serve as key coping mechanisms. While over half see no positive effects, 40% view increased family time as a positive consequence of the pandemic [92].

Regarding family as a protective factor, adherence to rules is crucial for parents’ happiness. Family relationships and friendships significantly contribute to children’s happiness, with correlations between children’s self-reported happiness, perceived parental happiness, and parenting styles [94].

Attachment security predicts lower student exhaustion symptoms and promotes adaptive emotion regulation strategies, mediating the relationship; no gender differences were identified. Through emphasising the importance of secure attachment, the study underscores the role of attachment security in encouraging adaptive emotion regulation strategies in children [89]. Online parenting programs help parents to manage children’s emotional needs, reducing emotional dysfunctions [95].

In regard to victimisation, school social support and climate are associated with the occurrence of dating violence victimisation among European adolescents. Creating a supportive school climate and utilising peer and teacher support are crucial in preventing dating violence [87].

During the COVID-19 pandemic, a significant number of education representatives identified increased instability within the educational environment. Information flows between the central, county, and local levels did not seem to function properly, especially regarding the transition to online scenarios or assistance with the diversity of available educational platforms [96].

Students express a preference for integrating electronic educational resources into face-to-face learning activities, citing the crucial role these resources played during the pandemic. The increased popularity of online materials that are accessible anytime and anywhere suggests practical recommendations for balancing face-to-face and digital education through hybrid learning. Despite the importance of in-person interaction, online learning’s accessibility and time-saving advantages lead to its growing role in the future [88,90].

Post-COVID-19, high school students have exhibited behaviour, health, and education changes, showing adaptability and resilience. Notable improvements include vaccination rates, mental health support perceptions, participation in health discussions, and overall mental well-being. Knowledge about contraceptives and quality of life has increased in the latter part of the pandemic [93].

When discussing prevention systems, the use of mental health strategies and implementing solutions involves preparing legal, financial, and educational support aspects. In order to effectively implement digital tools in depression prevention, public policies must address legislation, healthcare training, and public education, with a focus on digital skills and new technology acceptance [97].

Quarantine in Romania dramatically increased student inactivity, harming both their physical and mental well-being. Social isolation, lack of in-person schooling, and cancelled sports events all worsened the situation [98].

### 3.7. Sexual Education and Reproductive Health

According to the World Health Organization, comprehensive sexuality education (CSE) provides young people with accurate, age-appropriate information on sexuality and reproductive health (SRH), providing a foundation for making informed decisions about their health [99]. Reproductive health means having a safe, satisfying sex life, the ability to have children, and the freedom to choose if, when, and how often to do so [100].

In order to combat rising STI rates, adolescents must have access to comprehensive screening and treatment [101].

In this section, we examine 11 papers, two of which focus on adolescent pregnancy. One of them was a non-standardised questionnaire, and the other was a review using data collected from the Health for All European database and the United Nations Statistics Division Demographic Yearbook database. The nine other studies used questionnaires, two of which were standardised but used different standards. Seven of the studies used non-standardised questionnaires. One study performed interviews regarding the intention to vaccinate against HPV and seasonal flu. Another study performed interviews on sexual behaviour and health risks. Only one study was identified as an interventional study, consisting of a health education program based on lecture-based instruction with interactive follow-ups among adolescent girls in rural areas (Table 7). The extended data from the main findings of this section are detailed in Table S7 of the Supplementary Materials.

School programs should integrate family–adolescent relationships, considering the disparity between parental and school-based sex education. Striking a delicate balance is crucial; schools need to align with family, community, and societal norms in order to foster physically and mentally healthier generations [102]. In terms of sexually transmitted infections (STIs), effective preventive campaigns in Romania should be informed by a comprehensive understanding of young adults’ sexual knowledge, attitudes, and behaviours. Many students exhibit risky sexual behaviours and lack sufficient knowledge about STIs. Romania still has one of the highest rates of sexually transmitted diseases, especially among adolescents [103].

An examination of syphilis and gonorrhoea incidence among young people in Romania shows a decline in both infections. Syphilis is more prevalent in women and rural areas, while gonorrhoea is more common in men and urban areas. Overall, there is a decreasing trend in both syphilis and gonorrhoea among adolescents aged 15 to 19 [104].

In order to achieve global and Romanian HIV reduction goals, faster screening and follow-up care are crucial. Romanian physicians are interested in expanding screening and training to better address these goals [105].

Romania faces a high incidence of cervical cancer, constituting 7.5% of annual cases in Europe, with a mortality rate three times the EU average (14.2 per 100,000 women) [106]. A study on high-risk human papillomavirus (hrHPV) infection in Romania indicates ethnic and regional disparities, providing essential insights for targeted interventions [107].

Despite health education efforts, healthcare professionals struggle to inform adolescents and families about HPV’s negative effects and vaccination, requiring ongoing training. Barriers to vaccination need further exploration [108].

The adherence of the population to preventive programs, coupled with healthcare professionals’ efforts, is crucial. Schools, teachers, and school doctors can contribute significantly to educating about HPV infection. Informative campaigns involving professionals and tailored programs for adolescents are recommended [109,110].

Enhancing awareness of HPV infection is essential, particularly among medical students. Additional educational measures are needed in medical education [111].

For the success of national programs, Romania should organise sustained campaigns targeting both patients and doctors, ensuring continuous funding, simplifying bureaucratic procedures, and adopting gender-neutral HPV vaccination campaigns [112].

Unprotected sex, the early initiation of sexual activity, and inadequate education regarding sexual and reproductive health should be addressed in schools by experts and at home by parents. Effective prevention requires tailored programs for high-risk populations, including rural residents, those with a lower level of education, and young people [113].

**Table 7:** Included studies that address sexual education and reproductive health.

**Type of study**	Cross-sectional (n = 9)									Observational (n = 1)	Prospective (n = 1)
**Methods**	Questionnaire regarding sexual behaviour, attitudes, and knowledge about STIs, adolescent pregnancies, vaccination, and cervical cancer									Data collection from the sexually transmitted infections (STIs) surveillance system	Questionnaire, health education lessons
**Population**	12,395 median age 15 years from 11 European countries, including Romania (n not specified)	3872 aged 18–25 years	1363 aged 17–24 years	1359 people aged 18–30 years	736 aged 18–44 from 56 countries, including Romania (n not specified)	690 aged 18–19 years	100 aged under 18 years and 100 aged over 18 years	Girls under 20 (n = not specified)	401 aged 18–26 years	939 aged 15–19 years	534 aged 14–18 years
**Reference**	[114]	[103]	[109]	[115]	[111]	[110]	[116]	[117]	[118]	[104]	[119]
**Category of issues treated**	**Main findings of all studies (n = 11)**										**Frequency (percentage)**
Sexual behaviours	Age of first sexual contact often >15 years (38%); more common in boys (31.3%) vs. girls (16.9%)										1 Study (7.1%)
	Early sexual debut and multiple partners linked to lack of contraception use										1 Study (7.1%)
	Mental health issues associated with increased risky sexual behaviours, especially in younger students and females										2 Studies (14.2%)
Knowledge and attitudes	Awareness of HPV as a cervical cancer risk factor varies with gender, age, and ethnicity										2 Studies (14.2%)
	Knowledge gaps about specific STIs exist despite existing general awareness										1 Study (7.1%)
	Attitudes towards HPV vaccination differ; some lack knowledge of effectiveness										1 Study (7.1%)
	Fear of regret after inaction is a key motivator in the decision to get the HPV and flu vaccinations										1 Study (7.1%)
Information sources	Adolescents get sexual health info. from varied sources (doctors, internet, and family)										2 Studies (14.2%)
	Girls rely on family for STI info. but specific knowledge is limited										1 Study (7.1%)
Health outcomes	28% of births were to young mothers (13–18 years)										1 Study (7.1%)
	Temporary increases in teen pregnancy rates within a broader decreasing trend										1 Study (7.1%)
	Syphilis and gonorrhoea rates decreased, with differences in detection, gender, and location										1 Study (7.1%)
Other factors	Open family communication about sex linked to healthier sexual transitions										1 Study (7.1%)
	Young people often initiate SRH conversations with same-gender parents, triggered by milestones										1 Study (7.1%)

The colors represent the delimitation of the study: dark gray represents the first part of the table where I have type of study, methods, population and references displayed on vertical, and the light gray line referring to category of issues treated, main findings and percentage are for all studies listed up, listed on the horizontal.

Adolescents prefer in-school lectures for sexual education but find online sites most useful for STD information, with brochures being the least preferred source [119].

Additionally, if we are discussing pregnancy among adolescents, this remains a major global health issue affecting all countries, regardless of their income. Solutions are needed to prevent early pregnancy in this age group and, ultimately, to improve outcomes for both mothers and infants [120].

In Romania, high rates of teenage pregnancies are linked to limited information about sexuality and family planning at a young age. Particularly in rural areas, midwives could contribute significantly to addressing these issues if they are more involved in programs [121]. Despite a decrease in teenage pregnancy rates, the numbers remain notably high in Romania, with discrepancies in data reported by various databases [117].

Educational programs targeting both rural and urban schools and communities are essential to address teenage pregnancies. Poverty exacerbates the issue, leading to inadequate medical supervision, school dropout rates, illiteracy, and employment challenges. Midwives, through tailored health education, can play a vital role in rural communities, focusing on educating the parents of teenage mothers and improving communication with them [116].

Recognised links between mental health and adolescent reproductive health emphasise the need for interdisciplinary interventions considering age, gender, and behaviours [114].

Initiating discussions about sexual and reproductive health within families, often after significant events in a young person’s sexual life, is crucial. In designing health programs for adolescents, family involvement in sexual education is vital. This—delivered differently for boys and girls—may influence men and women to have sex at different ages. Additionally, parental anxiety about discussing sexuality correlates with a lower likelihood of discussing these topics with their children [115,122].

An educational framework and support for parents from adolescent psychology and psychiatry professionals would be highly welcomed for the harmonisation of sexual health education timelines and content between schools and families [123,124].

### 3.8. Access to Health Services and Preventive Medicine: Health Promotion and Disease Prevention

When discussing health education, the topics covered typically include nutrition, personal hygiene, physical activity, rest, mental health, reproductive health, family health, substance consumption/abuse, accidents, violence, and physical abuse [125].

In this section, we analyse six studies that used very heterogeneous designs and methods. One covers the health behaviours in school-aged children using a questionnaire method; one is a retrospective cohort analysis study regarding Roma population vaccination; one is a prospective study using a focus group of health professionals; one is a review study about health educational initiatives (Table 8). The extended data from the main findings of this section are detailed in Table S8 of the Supplementary Materials.

Recognising the crucial role of school medical systems in Romanian healthcare, urgent actions are proposed. The Ministry of Health should lead as the co-ordinating authority for national public health policies, defining school medicine’s position in primary healthcare, public health, and occupational health for pre-schoolers, students, and emergency medical care for students. Alternatively, forming a National School Medical Council with representatives from medical, educational, and administrative sectors is suggested. This council would develop essential strategies for optimal medical care in educational institutions [126].

School-based health screenings, specialist referrals, and rehabilitation measures are crucial for early disease detection and maintaining the health of children and youth.

School medical offices play a vital role in detecting treatable conditions, but need strong intersectoral collaboration and proper equipment to overcome challenges and improve student health outcomes [127].

A comprehensive analysis of Romania’s paediatric healthcare system reveals strengths such as free treatment for children under 18 and a basic vaccination program. However, the challenges include a shortage of medical staff, inadequate training in specific paediatric domains, and communication gaps among healthcare professionals. The studies emphasise the need for regular staff training, improved national health programs, and better health education for children [9].

Between 2000 and 2019, life expectancy at birth in Romania increased by over four years; however, in 2020, it decreased by 1.4 years due to the impact of the COVID-19 pandemic, maintaining one of the lowest positions in the EU. Behavioural risk factors contribute to over half of all deaths, with high rates of tobacco consumption, unhealthy eating, alcohol intake, and low physical activity reported by Romanians. Teenagers also exhibit high rates of overweightness, obesity, and smoking, which has been steadily increasing over the last two decades. Romania’s per capita spending on prevention is the second lowest in the EU, indicating insufficient resources and inefficiency in public health before the pandemic. Additionally, Romania’s spending on primary healthcare is the lowest in absolute terms among EU countries. Deficiencies in primary healthcare and prevention may explain Romania’s high rates of mortality from avoidable and treatable causes, ranking fourth in the EU in 2017 [128].

The barriers to broader paediatric palliative care in Romania include mental health support for providers, mobile service development, and the retention of qualified staff. Addressing these areas is crucial for sustainable paediatric palliative care in oncology [129].

Concerning vaccination, one study highlights the fact that unvaccinated paediatric patients of Roma ethnicity frequently suffer measles complications, particularly pneumonia. Addressing parental reluctance, especially within the Roma population with the lowest vaccination rates, is crucial. This provides an excellent opportunity for education to achieve complete eradication through vaccination [130].

Given insufficient vaccination coverage, research on the psychological factors affecting vaccine acceptance remains relevant. This study adds to the existing literature through exploring the predictive factors of intention to vaccinate against HPV and influenza in Romania, where vaccination rates are low [118].

Regrettably, the current pandemic has heightened vaccine hesitancy, with unique determinants coming to the forefront. Social media platforms, while used by the anti-vaccine movement to spread misinformation, also offer the potential for promoting health information and vaccination [131].

Religious service attendance and personal prayer are cumulatively linked to lower abortion acceptance, with young people being the most religiously devout and least accepting of abortion [132].

Adolescent health interventions should prioritise the specific needs of low-income youth, who report more health issues. This highlights the importance of addressing social inequalities for effective programming that really meets their needs [133].

There is a paucity of both empirical research and government analysis within the Romanian context that quantitatively assesses the efficacy or impact of health education initiatives.

We found some specific examples, such as a comprehensive analysis referring to the Review of 20 Years of Health Education Programs in Romanian Primary Schools [134]. This study talks about The National Program “Education for Health in the Romanian School”, which was launched in 2001 and was implemented (since 2004) as an optional discipline; it has been evaluated by an NGO (Save the Children Romania) that stated the impact of this has affected 6% of the child population in Romania [15].

**Table 8:** Included studies that address access to health services and preventive medicine in terms of health promotion and disease prevention.

**Type of study**	Cross-sectional (n = 2)		Literature review (n = 2)		Retrospective cohort analysis (n = 1)	Prospective (n = 1)
**Methods**	Questionnaire on health behaviour, human rights, abortion, euthanasia, and vaccines		Literature search on vaccine intentions, social media, immune system, hesitancy reasons, and implementation of the health education programs		Data from clinical and laboratory records from patients treated for infectious diseases	Focus groups with healthcare providers and patient representatives from multiple paediatric oncology centres across Romania
**Populations**	228,979 aged 11–15 years from 45 countries, including Romania	987 aged 14–18 years		-	104 under 10 and 32 between 10 and 18 years	-
**Reference**	[133]	[132]	[131]	[134]	[130]	[129]
**Category of issues treated**	**Main findings of all studies (n = 6)**					**Frequency (percentage)**
Health disparities	Distinct profiles of health complaints, with low SES (socio-economic status) linked to greater physical and psychological issues					1 Study (12.5%)
	Roma minority over-represented in the study, highlighting potential health disparities					1 Study (12.5%)
Access to care	Barriers to implementing paediatric palliative care: emotional demands, work-life balance, staffing, financial limitations, stigma, and political instability					1 Study (12.5%)
Health conditions	Children under 10 are more likely to contract an infection within the family compared to adolescents					1 Study (12.5%)
	Risk factors for pneumonia: age, nutrition, Roma ethnicity, anaemia, and procalcitonin levels					1 Study (12.5%)
Preventative care	Vaccination decisions differ between adolescents and parents and are complex for children with neurological disorders					1 Study (12.5%)
	Limited quantitative research and evaluation of health education initiatives in Romania					1 Study (12.5%)
Attitudes and beliefs	Religiosity (church attendance and prayer) influences attitudes on euthanasia, abortion, and socio-economic rights					1 Study (12.5%)

The colors represent the delimitation of the study: dark gray represents the first part of the table where I have type of study, methods, population and references displayed on vertical, and the light gray line referring to category of issues treated, main findings and percentage are for all studies listed up, listed on the horizontal.

Another program, the “National Health Strategy 2014–2020, Health for Prosperity”, provides evidence supporting the need for health education in Romanian pre-university schools. This framework document was prepared by the Presidential Administration, UNICEF Romania, the Ministry of National Education, the Ministry of Health, the World Health Organization, the National Institute of Public Health, and the National School of Public Health Management and Improvement in the Health Field. The authors of this document did not provide the guidelines for its implementation. Schools lack consistent health education programs and must provide age-appropriate frameworks tailored to student needs [134].

The study highlights the lack of standardised health education programs in Romanian schools. While large-scale initiatives, such as “Healthy Choices” by Save the Children, address important topics (nutrition, physical activity, mental health, sex education, and substance abuse prevention), these are not uniformly implemented. Smaller school-based projects often focus on nutrition, environmental health, hygiene, and healthy habits but lack impact assessment [134].

As mentioned in the introduction, a law regarding healthcare reform was passed in 2022, which includes concrete data on how health education can be implemented in Romania; however, guidelines for its practical use are still required [16]. In order to address the disadvantages faced by young Romanians, policy interventions must go beyond education. While education plays a crucial role in income distribution, a holistic approach is necessary. This includes considering factors such as gender, urbanisation, region, industry, and work experience [135].

## 4. Discussion

This narrative review study is the first of its kind from Romania, covering a wide range of topics and gathering and correlating information to analyse the current state of health habits, healthy choices, and health education among children and young people in Romania.

Some of the factors identified in the study that influence the healthy choices of children and young people are socio-economic status and parental education, indicating that less educated parents facilitate children eating less healthy food, spending too much time on a phone, social networks, or computers (playing games), practising fewer sports, and smoking or drinking. Moreover, children’s oral hygiene and health status are associated with parental education and economic status [136]. Paediatricians, as primary healthcare providers, play a crucial role in children’s oral healthcare. They should possess the knowledge to provide anticipatory guidance and dental education to parents, supporting informed decision-making [137].

When discussing nutrition, the obesity rates among young people have been increasing in Romania in recent decades, and the lack of education in schools on this topic is relevant, as this is a field that has been evidenced in other countries as significantly helping increase good health choices. A Croatian study demonstrates the positive medium-term impact of education-based programs on the nutrition knowledge, diet quality, lifestyle, and overall health of 10–12-year-old schoolchildren [138]. Another recent review of the nutritional status of European children and adolescents shows that the most significant nutrition issues are overweightness and obesity [139]. Childhood obesity can lead to serious cardiovascular health problems in adulthood, potentially causing premature death or early disease [40]; therefore, reducing obesity rates should be one of the main focuses of our health policies, including campaigns and formal and non-formal education programs for children and their parents.

The prevalence of eating disorder (ED) issues in children and adolescents represents a significant concern that has not been fully addressed within the national context. Factors such as social media influence and widespread societal concerns over obesity and body image contribute to the escalation of EDs. These influences often result in the underdiagnosis of EDs, individuals using ED specialists, and psychiatric professionals being required at more severe stages. It is imperative to adopt a proactive and informed stance on the issue for early detection and interventions in the incipient phases and intervening strategies to mitigate the impact of societal pressures on the youth [140].

The same factors are also related to alcohol consumption and smoking. In central and eastern European countries, tobacco use susceptibility among young people is strongly correlated with social, educational, and political factors, as well as attitudes towards tobacco use [67]. A particular target issue would be the status of any inhaled substances in the tobacco class (e-cigarettes) that have managed to sidestep all the regulations in force regarding tobacco; these are erroneously perceived by most of the population as non-harmful, triggering a smoking epidemic among teenagers [71,141]. A systematic approach to the smoking issue—which has a high prevalence in Romania—is mandatory in order to come closer to achieving the EU objective of less than 5% of people using tobacco by 2040 [142]. The WHO states that there is no safe level of alcohol consumption, as any amount poses health risks due to the harm caused by alcohol itself [143].

A rise in mental health problems among Romanian adolescents is part of a broader European trend, according to the latest Health Behaviours in School-aged Children (HBSC) report by the WHO. The report delves into the mental health status of adolescents across Europe, considering gender, age, social disparities, and temporal trends [144].

Concerning sexual education and health education, a risk factor is the lack of programs in schools, the lack of information, and the lack of strategies and health policies, which place Romania first in Europe for HPV-related cervical cancer and teenage pregnancies. Many countries from Europe have developed their programs on this topic with very good results, as highlighted by a study investigating the risk and protective factors associated with adolescent health behaviours in a European context [145].

Adolescents are open to discussing personal health and prefer modern tech for gathering information, but they trust dentists, schools, and family the most for oral health problems. They also enjoy group debates on familiar topics [30]. There are Romanian studies that highlight systemic shortcomings in addressing student health education needs. This necessitates a comprehensive approach integrating physical activity promotion, technology, substance abuse prevention, and harassment intervention [58,66,91]. The implications of the study findings are to be considered by public health policies in Romania. These research outcomes can inform policy decision-makers to form a good image of children and young people’s health.

Future research needs to be performed on these topics. The current study identified some gaps in the information regarding the highlighted areas; we considered topics—such as education outcomes and how the interventions from education programs can have an impact—that could further enhance understanding of the health-related behaviours and factors in this demographic, when contrasted with the official reports we generally receive about the healthy choices of children and adolescents.

This study’s findings contribute to the global understanding of children and young people’s health. We consider the extrapolation of these results to other contexts with similar socio-cultural backgrounds and risk and protective factors, such as urbanisation and lifestyle patterns, healthcare system differences, and accessibility between rural and urban areas or cultural specificity.

### Limitations

This study’s findings may be limited due to the use of only three databases and a focus on open-access articles. Despite these limitations, we believe that, although it is true that expanding our search to include more databases might have uncovered additional studies, the overall scarcity of studies in this research area in Romania suggests that the associated impact on our findings might be minimal.

Romanian papers were also included. As a literature review, this study did not include a statistical analysis of the data.

This review did not explore the impact of a missing national health education strategy on the healthcare system.

## 5. Conclusions

The literature unequivocally highlights a disparity in the adoption of healthy lifestyle attitudes among children and young people in Romania. This underscores the need to implement health education programs focusing on healthier behaviours and lifestyles.

Despite strides in various sectors, Romania confronts formidable hurdles in ensuring equitable and high-quality healthcare services for its youth.

Vital aspects such as nutrition, immunisation, and access to essential medical services stand out as pivotal focal points for enhancing the well-being of Romania’s young population. Addressing these areas comprehensively is imperative, in order to safeguard the health and future prospects of Romania’s children and young people nationwide.

The advent of the COVID-19 pandemic exacerbated the existing vulnerabilities, particularly in the field of mental health among children and young individuals. Efforts to ameliorate this crisis demand sustained commitment and the provision of adequate support systems to mitigate the pandemic’s enduring effects on mental well-being.

It is abundantly clear that health education and the promotion of healthy lifestyles remain indispensable strategies for bolstering the health outcomes of Romania’s youth. Through targeted interventions and community-wide initiatives, concerted efforts can be made to empower children and young people with the knowledge and tools necessary to adopt healthier behaviours.

## Figures and Tables

**Table 2:** Included studies that address eating habits and nutritional status.

**Type of study**	Cross-sectional (n = 8)								Literature review (n = 2)		Descriptive observational (n = 2)		Prospective (n = 1)
**Methods**	Questionnaire about lifestyle and eating habits; anthropometric measurements								Review of the existing literature from the year 2000 onwards on available local data about paediatric obesity		Clinical examination with anthropometric data, skin examination (acanthosis nigricans, hirsutism, and striae), physical exam, electro-cardiography and echo-cardiographic exams; questionnaire on lifestyle, eating habits, and food behaviours		Clinical examination of age, sex, anthropometric measures, and BP (blood pressure) value
**Populations**	21,650 aged 7–18 years	880 aged 10–15 years and 665 parents	866 aged 6–18 years	530 aged 1–16 years (2 groups: Romanian children living in Romania (RCR); Romanian children living in Italy (RCI)	344 children aged 11–14 and 147 parents	328 aged 2–18 years	285 aged 11–14 years	60 aged 6–18 years	Total cohort (n = 36, 048) aged 2–18 years	Total cohort (n= 25,060) aged 6–19 years	1165 aged under 10 years and more than 10 years	251 aged 7–17 years	335 aged 2–18 years
**Reference**	[34]	[35]	[36]	[37]	[38]	[39]	[40]	[41]	[42]	[43]	[44]	[45]	[46]
**Category of issues treated**	**Main findings of all studies (n = 13)**												**Frequency (percentage)**
Dietary intake	Romanian children in Italy had healthier diets than those in Romania (KIDMED Index)												1 Study (6.66%)
	RCR had lower fast food/sweets consumption and higher nuts/yoghurt/cheese intake												1 Study (6.66%)
Eating behaviours	Skipping breakfast was common, especially in girls												1 Study (6.66%)
	Eating in front of screens is common												1 Study (6.66%)
	Most children met physical activity recommendations												1 Study (6.66%)
Weight status	Overweight/obesity prevalence varies with age and classification system (22.3–31.6%; 6.2–12.5%)												1 Study (6.66%)
	Underweight prevalence ranges from (2.6–6%)												1 Study (6.66%)
	Boys have higher overweight/obesity prevalence than girls												1 Study (6.66%)
	10-year-olds had the highest overweight/obesity prevalence; 18-year-olds had the lowest												1 Study (6.66%)
	Children are developing weight-related health conditions (hypertension, dyslipidaemia, etc.)												1 Study (6.66%)
Perceptions	Parents often misperceive their child’s weight status, especially underweight and overweight												1 Study (6.66%)
	Children with weight misperceptions are most likely underweight or overweight												1 Study (6.66%)
Correlations	Unhealthy eating behaviours are common but not directly correlated to obesity/overweight alone												1 Study (6.66%)

The colors represent the delimitation of the study: dark gray represents the first part of the table where I have type of study, methods, population and references displayed on vertical, and the light gray line referring to category of issues treated, main findings and percentage are for all studies listed up, listed on the horizontal.

## Data Availability

Not applicable.

## Supplementary Materials

The following supporting information can be downloaded at https://www.mdpi.com/article/10.3390/medicina60050725/s1: Tables S1–S8: Supplementary materials—detailed findings of the studies (Tables S1–S8). Supplementary Table S1. Included studies that address oral health. Supplementary Table S2. Included studies that address eating habits and nutritional status. Supplementary Table S3. Included studies that address physical activity. Supplementary Table S4. Included studies that address alcohol consumption. Supplementary Table S5. Included studies that address the use of tobacco and tobacco-like products. Supplementary Table S6. Included studies that address the mental health and well-being situation (both before and after the COVID-19 pandemic) and online activities. Supplementary Table S7. Included studies that address sexual education and reproductive health. Supplementary Table S8. Included studies that address access to health services and preventive medicine in terms of health promotion and disease prevention.
