# Supplementary material for: Status of Healthy Choices, Attitudes and Health Education of Children and Young People in Romania—A Literature Review
Source: Medicina (Kaunas). 2024 Apr 27;60(5):725. doi: 10.3390/medicina60050725 (PMC11123286; doi:10.3390/medicina60050725)
Supplement: Supplementary file 1 [file medicina-60-00725-s001.zip › medicina-2905085-supplementary.pdf]

SUPPLEMENTARY MATERIALS – DETAILED FINDINGS OF THE STUDIES (Tables 1-8)

Supplementary Table 1. Included studies that address oral health.

Author, Year	Type of study	Population	Method	Main findings
Dumitrescu R et al, 2022 [22]	Prospective study	809 children aged 6 to 8 years	Questionnaire "Romanian Oral Health Survey"; Clinical oral examination	- This study found a high average of decayed, missing, or filled teeth (DMFT value = 4.89) and a high Significant Caries Index (SiC = 9.83). Importantly, a child's DMFT score was negatively associated with both their father's and mother's level of education
Tudoroniu C et al, 2020 [23]	Cross-sectional study	650 adolescents aged 10 to 19 years	Questionnaire on dental behaviour section and a validated food frequency questionnaire (FFQ). Clinical oral examination	- The study revealed an exceptionally high prevalence of dental caries among adolescents, with 95.5% affected<br>- Average DMFT was similar between rural and urban adolescents, but lower in females than males ($p < 0.050$)<br>An alarming 33.7% of adolescents don't brush their teeth in the evening, or do so very infrequently<br>- Skipping regular dental checkups significantly increases the risk of tooth decay, with 40.6% of adolescents missing these important appointments<br>- This study reveals two significant factors contributing to tooth decay: age ($p = 0.020$) and consuming sugary sweetened beverages ($p = 0.028$)
Saveanu CI et al, 2022 [24]	Cross-sectional study	718 adolescents aged 10 to 19 years	Questionnaire on Oral Hygiene Knowledge and Attitudes	- The majority of the students understand basic toothbrushing techniques and that brushing removes plaque.

				However, far fewer know that brushing also helps remineralize teeth<br>- Toothbrushing duration varies significantly among participants, with only a weak correlation to the recommended 2-3 minutes<br>- Participants reported that dentists' recommendations were the primary factor in toothbrush selection, while toothpaste choices were influenced by perceived product properties<br>- Most participants brush twice a day; mouthwash is utilised after each brushing to the majority of them with a significant less proportion of using dental floss.
Sava-Rosianu R et al, 2021 [25]	Cross-sectional study	814 adolescents aged 11 to 14 years	Questionnaire on WHO Oral Health Surveys—Basic Methods, 5th edition; Clinical oral examination	- The average dentinal caries index was 4.96, but there was significant variation among individuals (SD = 5.33)<br>- Girls exhibited a higher likelihood of having non-zero restoration codes<br>- Children with parents who have lower education levels were more likely to have cavities ($\beta = 0.23$, SE = 0.06, $p = 0.01$; $\beta = 0.22$, SE = 0.06, $p < 0.01$)<br>- Consuming carbonated soft drinks, candies, sweetened milk, tea, or cocoa was significantly associated with an increased likelihood of cavities<br>- Children in rural areas were more likely to have cavities. Additionally, there was a negative correlation between a county's development index and the number of dental fillings/restorations, suggesting that children in less developed counties have fewer restorations.
Dumitrescu R et al, 2022 [26]	Cross-sectional study	814 children aged between 11 to 14 years	Questionnaire on WHO Oral Health Surveys-Basic Methods, 5th edition, 2013	Children of mothers with higher education levels were more likely to have good oral hygiene habits (regular checkups, brushing, use of hygiene aids) and less likely to experience tooth pain

				- Children of mothers with higher education were more likely to have dental fillings<br>Children of fathers with higher education levels had better oral hygiene habits but were also more likely to have decayed, missing, or filled teeth (D3T index)
Sfeatcu R et al, 2023 [27]	Cross-sectional study	98 children aged 12 years	Questionnaire on Oral Health Questionnaire for Children Organization; clinical examination, using International Caries Detection and Assessment System (ICDAS)	- While 36.7% of 12-year-olds were free of tooth decay, the average DMFT (indicator of dental health) was 2.89<br>Despite some positive habits, like 38.8% of children brushing twice daily, there are concerning trends: frequent consumption of sugary foods and 11.2% of 12-year-olds never visiting a dentist
Ilici RR et al, 2019 [29]	Longitudinal interventional study	76 adolescents<br>Between 12-16 years	Questionnaire; Clinical oral examination; Intervention: The test group participated in hands-on learning experiences, while the control group received a standard toothbrushing demonstration	After the educational lessons, children of both ages brushed their teeth correctly more often.<br>-The educational intervention did not result in a reduction in the frequency of cake and biscuit consumption for either age group<br>-Adolescents of 15–16-year-olds are drinking fewer carbonated drinks daily

				-Initially, only about a third of students understood the benefits of fluoride, but the educational intervention significantly increased this knowledge – especially in the older age group, where all students understood its role afterward, and on the other group were more than a half.<br>- Almost all students (over 90%) didn't know proper toothbrushing techniques before receiving oral health education.
Chisnoiu RM et al, 2022 [30]	Cross-sectional study	258 patients aged between 16 and 69 years	Questionnaire; Clinical oral examination	- Most patients perceive their oral health to be moderate or good. Notably, females are more likely to report poor oral health compared to males.<br>- Most participants (67%) learned about oral health from their families and dentists during childhood<br>- The vast majority of patients (90%) understand that good oral health requires brushing, limiting sugary foods, and regular dental visits<br>- A significant majority of patients (69.7%) place high value on oral health<br>- This study identifies high treatment costs and fear of dental procedures as the most significant factors deterring patients from visiting the dentist.<br>While nearly a quarter of people visit the dentist regularly, for the majority (75%), pain remains the primary reason to seek dental care<br>- Nearly half (48%) of patient's report brushing their teeth twice a day and supplementing their routine with mouthwash<br>- Approximately 50% of participants indicated that their eating habits include daily consumption of sweetened foods or drinks

				- 75% of patients had dental problems, including calculus buildup and evidence of tooth decay or extractions at clinical examination
Graça SR et al, 2019 [31]	Cross-sectional study	879 adolescents, aged between 12-16 years, from three countries, Romania (n = 455),	Questionnaire<br><br>On adolescents' perceptions and habits of oral health	Reported sources of information: - the dentist 53.6%; - the dental hygienist 10%.<br><br>- Professional advice: - toothbrushing (68%); caries prevention; dental floss use (25,7%), disease prevention (27.4%)<br><br>- no advice received: 12%<br>- The lowest rate of advice on flossing was received by Romanian adolescents (P < 0.001)<br>- Periodontal diseases prevention through toothbrushing was unknown to 74.9% of the responders<br>- While 55.9% identified energy drinks as harmful for teeth, 86.5% responded that sweet drinks can affect oral health<br>– 40.5% identified fluoride as beneficial for teeth protection from caries; 27.3% were unaware of what it is<br>Romanian and Swedish adolescents' oral health knowledge was similar while Portuguese adolescents ranked higher.
Podariu AS et al, 2017 [142]	Interventional study	739 adolescents aged between 11-16	Evaluation of oral health, oral health lessons, follow-up at two years	-Following-up after two years indicates a significant statistical decrease of caries, gingivitis, and malocclusion incidence<br>-Fluoride toothpaste use twice daily ↑ from 568 to 663 participants; dental sealants ↑ from 182 to 514.<br>- Sugary food consumption as snacks ↓ from 418 to 279, and drinks ↓ from 464 to 413<br>– Correct toothbrushing technique ↑ from 482 to 578; - Correct flossing technique ↑ from 268 to 568<br>- Oral breathing and narrow maxilla were ↓from 303 to 182. Low and forward tongue posture ↓ from 406 to 289 participants.

SUPPLEMENTARY MATERIALS – DETAILED FINDINGS OF THE STUDIES (Tables 1-8)

Supplementary Table 2. Included studies that address eating habits and nutritional status.

Author, Year	Type of study	Population	Method	Main findings
Rădulescu C et al, 2020 [34]	Literature review	total cohort n= 36 048 aged 2-18 years	Review of existent literature from the year 2000 and onwards, on available local data about paediatric obesity.	- A significant proportion of children are overweight or obese (18 - 31%).<br>– Children are beginning to develop diseases such as hypertension, dyslipidaemia. The impact is still difficult to assess.<br>- Children aged 2-19 years are: 23.77% excess weight, 16.6% overweight, 8,7% obese
Luca AC et al, 2022 [35]	Retrospective descriptive study	1165 between under 10 - more than 10 years	Clinical examination with anthropometric data, skin examination (acanthosis nigricans, hirsutism, striae), physical exam, electrocardiography, and echocardiographic exams.	- hypertension 15.36%, concentric hypertrophic cardiomyopathy 11.15%, atherosclerosis risk 13.04%, hypercholesterolemia 20.94%<br>- Epicardial adipose tissue thickness and diastolic dysfunction ↑ obese children, not connected to hypertension or coronary impairment<br>- Diastolic dysfunction of the left ventricle (whole group), ventricular hypertrophy 21.8%, hypertrophic cardiomyopathy 11.15%.
Miron VD et al, 2021 [36]	Prospective study	335 aged 2 - 18 years	Clinical examination on age, sex, anthropometric measures, and BP (blood pressure) value.	-3.4 Increased risk of having high BP values was associated with children overweightness<br>- 60.9% were normal-weight), and 26.0% children were overweight or obese<br>- 29.3% of participants had BP values above the 95th percentile.<br>-Patients’ age or gender were not statistically corelated with obesity or high BP

				- 49.4% of overweight or obese children, had higher than normal BP values
Chirita-Emandi et al, 2016 [38]	Literature review	total cohort n= 25,060 aged between 6-19 years	Review of studies between 2006 and 2015, classifying BMI WHO, IOTF, and CDC references.	- Underweight children's prevalence: 4.5-8.5%<br>-Overweight prevalence remains constant over the last ten years (chi-square test p = 0.6).<br>- -High risk for overweight: male gender compared with female gender, prepuberal to postpuberal age and urban to rural areas.
Negrut GA et al, 2022 [39]	Cross-sectional study	328 aged 2-18 years	Questionnaire on the eating habits of children in Romania	- 63.11% -in the study were normal weighted<br>A significant percentage of mothers (82.32%) viewed their children as having a normal weight<br>- Mothers' perception on their children weight status was different to reality as 8.54% of children were obese, and only 1.22% of mothers considered this.<br>- -Obesity prevalence ↑ in children with low physical activity and high fastfood consumption.<br>– Parents' subjective opinion on children' weight is a contributing factor to become overweight or obese as a child.
Barbu GC et al, 2015 [40]	Cross-sectional study	866 aged between 6-18 years	Questionnaire about lifestyle and eating habits;<br>Anthropometric measurements	- Overweight prevalence: 22.3-31.6% (WHO, IOTF, CDC, local references)<br>- Obesity prevalence: 6.2-12.5% (same references)

				- Overweight prevalence: boys > girls, age group 6-10.9 years >11-17.9 years<br>- At least one unhealthy eating behaviour was reported by 95% of respondents, with no significant correlation with obesity or overweight alone.
Pop TL et al, 2021[41]	Cross-sectional study	21650 children aged 7-18 years	Following WHO guidelines, researchers collected weight and height data to calculate Body Mass Index (BMI) and its corresponding z-scores.	Rates of overweight and obesity differed by age and the classification system used (WHO, CDC, IOTF). Across all standards, 10-year-olds had the highest prevalence, while 18-year-olds had the lowest. Underweight was less common, with rates ranging from 2.6% - 6% depending on the standard<br>While overall rates of overweight and obesity were similar across reference systems (WHO, CDC, IOTF) in urban school-aged children, differences emerged when classifying specific categories: IOTF identified the highest rates of overweight; WHO standards found the highest rates of obesity and severe obesity.
Motoc GV et al, 2023 [42]	Cross-sectional study	60 aged 6- 18	Clinical consult on oral cavity with the micro-IDent test kit;<br>Anthropometric measurements	Both P. intermedia and B. forsythus infections were most strongly associated with normal BMI (50%). Additionally, P. intermedia was equally associated with being overweight (50%).<br>Overweight BMI was associated with infections of P. micros, C. rectus, and E. corrodens, while P. micros was also strongly linked to underweight BMI<br>The study found complex associations between BMI and bacterial infections: P. micros was linked to both underweight and overweight, while C. rectus and E. corrodens were associated with normal and overweight BMI

SUPPLEMENTARY MATERIALS – DETAILED FINDINGS OF THE STUDIES (Tables 1-8)

Pantea Stoian et al, 2018 [43]	descriptive observational study	251 aged 7-17 years	Questionnaire on<br>Lifestyle, eating habits, food behaviours	Boys had significantly larger waist circumferences than girls, and this was reflected in higher rates of overweight and obesity (27% in boys vs. 22% in girls)<br>This study reveals a significant gender difference in breakfast habits: 36.3% of girls skip breakfast, while most boys (63.8%) bring a snack to school<br>Nearly a quarter of both boys (23.8%) and girls (24%) report eating in front of screens
Trandafir AV et al, 2022 [44]	Cross-sectional study	880 children aged 10-15 years and 665 parents	Questionnaire about weight, eating habits, physical activity, sleep, bullying and cyberbullying;<br>Anthropometric measurements during school activities.	Among the children population, 61.0% exhibited normal weight classification. A concerning 7.4% presented as underweight, while 31.6% fell into the overweight or obese categories<br>While most parents correctly identified the weight status of their normal-weight children, only 30% accurately assessed their underweight or overweight children.<br>Several factors contributed to weight misperceptions, including body mass index (BMI), gender, weight-related behaviours, parents' weight estimations, family discussions about weight, and experiences with bullying (physical and online) and social exclusion.
Lotrean LM et al, 2021 [45]	Cross-sectional study	344 children aged 11 - 14 and 147 parents	Questionnaire about weight, eating habits, physical activity, sleep, bullying and cyberbullying;<br>Anthropometric measurements	The majority of children (68.3%) had a normal weight. However, a small percentage were underweighted (3.8%), and over a quarter were overweight (27.9%)<br>Children with misperceptions were most likely to be underweight (61.5%), followed by overweight children (43.7%). Only 20% of normal-weight children had misperceptions.

			during school activities.	Parents had the highest misidentification rates for underweight children (50%) followed by overweight children (41.5%), and normal-weight children (11.9%)
Năsui BA et al, 2023[46]	Cross-sectional study	285 aged 11-14 years	Questionnaire on lifestyle factors, dietary habits, and nutritional status (using BMI measurements)	This study shows that boys are at a significantly higher risk of being overweight or obese ($p = 0.001$).<br>- Children slept an average of 8.12 hours per night, with boys sleeping longer than girls<br>- Most respondents met age-appropriate physical activity recommendations, primarily through unstructured activities<br>-The dominant screen time among respondents was found to be 4-6 hours or longer.<br>Participants favoured comfort foods over vegetables and consumed less fast food
Pira C et al, 2021 [49]	Cross-sectional study	530 aged 1–16 years<br><br>(2 groups – Romanian children living in Romania (RCR) and Romanian children living in Italy (RCI)	Questionnaire - an adapted version of the KIDMED Test (Mediterranean diet quality index for children and teenagers)	Romanian children living in Italy had significantly higher KIDMED index scores (68.09) compared to those living in Romania (17.76), indicating healthier dietary habits ($p < 0.05$)<br>- RCR had healthier dietary habits in several key areas, explaining their higher KIDMED scores: lower consumption of fast food and sweets; increased consumption of nuts, yogurts, and cheese

Supplementary Table 3. Included studies that address physical activity.

SUPPLEMENTARY MATERIALS – DETAILED FINDINGS OF THE STUDIES (Tables 1-8)

Author, Year	Type of study	Population	Method	Main findings
Musić Milanović S et al, 2021 [51]	Cross-sectional study	124 700 aged 6 - 9 years from 24 countries, including Romania (n=9,094)	Questionnaire based on COSI design (Childhood Obesity Surveillance Initiative);<br><br>Anthropometric measurements	- Home to school transportation use is ↑ on high SES (SES - socioeconomic status) children based on parents possessing higher levels of education 45.6%, parents engaged in higher levels of employment 43.8%, those from families perceived as having greater wealth 41.3%<br>- Low SES children ↓ sports club participation; ↑ over 2 h screen time per day<br>-The level of parental education significantly impacts the type of sports activities children engage in: those with parents having lower education levels tend to spend less time on sports activities, with 70.9% engaging in less than 2 hours per week, whereas children with highly educated parents show a lower percentage, with 38.2% spending less than 2 hours per week on sports.<br>- The correlation between SES and sleep patterns showed variability depending on the specific indicator of SES being considered.<br>- Low SES doesn't consistently correlate with a higher prevalence of "less healthy" behaviours.<br>- Children with moderate parental education levels were 1.30 times more likely to engage in sports for less than 2 hours a week compared to children with high parental education levels. Those with low parental education levels were 2.24 times more likely to do the same.<br>- This indicates that each decrease in parental education level significantly raises the risk of lower sports engagement among children.
Popa PS et al, 2023 [52]	Randomised control trial	173 between 6-17 years	clinical examination - OHI-S (Oral Hygiene Index-Simplified), PMA (Papillary-Marginal-Attached Gingival Index), and DMF-T values were determined	- Contrary to findings in studies focusing on adult athletes, competitive sports have been shown to have a positive impact on the oral hygiene, periodontal health, and dental integrity of children.<br>- The performance sports groups exhibited lower (better) values across all three indices compared to the control group.

				- Oral hygiene, as assessed by the OHI-S index - mean value of 2.435 in the control group; hockey participants - mean value of 0.9797; football participants - mean value of 1.472. These results indicate better oral hygiene among individuals who regularly participate in physical activities such as hockey and football.<br>- Lack of identified studies specifically analysing the impacts of competitive sports on oral hygiene, periodontal health, and dental integrity among children who are athletes.
Druica E et al, 2021[53]	Cross-sectional study	665 aged 18–23 years	Questionnaire based on YPAP (Youth Physical Activity Promotion) framework	- All the theoretical dimensions of YPAP (predisposing, enabling, and reinforcing) exhibit a positive and significant influence on physical activity. Among these, two mediating mechanisms, expressed as predisposing factors—namely, ability and perceived worth—play a crucial role.
Ciucurel C et al, 2023 [54]	Cross-sectional study	124 aged 18-27 years	Questionnaire based on International Physical Activity Questionnaire—Short Form; clinical measurements of ECG	The exercise routine of young adults has a notable impact on the synchronization between ventricular depolarization and repolarization, which is evident in the width of the FQRST complex.<br>In healthy young adults, a narrow FQRST is linked to a high PAL, whereas in sedentary individuals, the angle tends to increase but typically remains within the normal upper limit.<br>Significant regression model relating BMI to the FQRST.
Anton-Păduraru DT et al, 2021 [55]	Cross-sectional study	1320 aged 14-18 years	Interview face-to-face after developing a questionnaire	- > 70% of participants were within the normal weight range.<br>- 63.56% elated 3–4 meals/day<br>- Breakfast was reported as the most frequently skipped meal -60.53%<br>- Consumption of grains, dairy products, and meat: boys > girls<br>- Consumption of vegetables and fruits: boys = girls.<br>- Servings of both fruits and vegetables were inadequate in the diets of a significant portion of adolescents, accounting for 39.31%.<br>- Daily alcohol consumption (2+ drinks) was reported by 5.68% of participants, with 37.87% consuming caffeine products one or more times per week

				- Perception on prevention of obesity: 95.2% of boys engaged in regular physical activity and 97.2% of girls discussing about the role of the right nutrition.<br>- In the study of the self-perception profile, it was found that 89.8% of boys and 79.5% of girls perceived themselves as having attractive bodies.<br>- Based on BMI classifications, 86.7% of normal-weight adolescents and 84.8% of underweight adolescents perceived themselves as having attractive bodies. In contrast, only 73.5% of overweight and 56.7% of obese adolescents considered themselves as such.
Ching Moc MM et al, 2020 [143]	Interventional study	3036 aged 8–11 years from 8 countries, including Romania	Questionnaire based on The Attitudes toward Physical Activity Scale (APAS); intervention - exercise videos provided by HOPSports Brain Breaks®	-The study reaffirmed the beneficial influence of exercise videos on the learning process<br>- Education constitutes an ongoing process, encompassing the exchange of curricular subject matter during classes and non-curricular or informal educational activities during recess or physical activity periods.

Supplementary Table 4. Included studies that address alcohol consumption.

Author, Year	Type of study	Population	Method	Main findings
Năsui BA et al, 2015 [60]	Cross-sectional study	468 aged 18-25 years	Questionnaire on alcohol consumption	- Alcohol consumption/ week: males > females<br>- Abstinence prevalence: males 10.8%; females 17.6%<br>- Individuals who frequently consume large amounts of alcohol (≥5 drinks, more than once/week) : males 19.3%, female 16.2%<br>- More often than not, drinking behaviours are associated with academic performance, but the potential connection between poor academic performance and alcohol

				consumption seems weak and warrants additional investigation.
Năsui BA et al, 2021 [61]	Cross-sectional study	722 aged 18-25 years	Questionnaire<br>on alcohol consumption	- At risk drinkers on medical students: 15% males, 14.9% females<br>- Illicit drugs consumption: male > female;<br>-Physical activity: male > female<br>- The group of female drinkers at risk consumed more drugs compared to the group of female drinkers at low risk.<br>-Engaging in additional risky behaviour linked to drinking including smoking, experiencing low academic performance, and driving a car after consuming alcohol, was observed in females and males drinkers.
Năsui BA et al, 2021 [62]	Cross-sectional study	1212 aged 18-25 years	Questionnaire<br>on alcohol consumption	- Alcohol consumption's prevalence on students: 79.9%<br>- Analysis of multiple regression revealed a positive association between drinking and gender, physical activity, smoking, and eating fast-food, and a negative association between drinking and sleep duration and study hours.<br>- Males in the study had a 18% prevalence on binge drinking<br>- Alcohol use correlates also with the regular use of illicit substances, but at a low prevalence rate (1.6%).
Jimborean MA et al, 2021[63]	Cross-sectional study	1044 aged 18-24 years	Questionnaire on alcohol consumption	A statistically significant correlation exists between the age of initial alcohol consumption and the presence of a family member with alcoholism.<br>- The primary motivations for alcohol consumption include taste, sensory experience, relaxation, and social interaction.<br>- Occasionally drinking in public places is preferred for males and females in the study.

				- Females exhibit a preference for sweet flavours, often opting for specialty beers over regular ones. Also, the choice of specialty beers among the students can be influenced by their place of residence.
Petrelli F et al, 2018 [145]	Cross-sectional study	1727 aged 17 years	Questionnaire on lifestyle	- 37.4% of students admit to smoking, with the habit typically starting between the ages of 12 and 15 - 41.3%, notably including 42.5% female students.<br>- 67% of students admit drinking, with the habit typically starting between 14-15 years.<br>- 92% of the responders claim that they abstain from drug usage. .<br>- Knowledge about the dangers on health related to drug usage was limited among students – 49.8% didn't knew the effect of amphetamine, 44.5% on methadone, 43.1% LSD, 43.8% steroids, 55.6% creatine.<br>- Beer consumption: 81.3% males, 66.5% females, wine consumption: less frequent<br>- Spirits consumption: males - 40.8%, females – 28.6%<br>- Situation of drinking spirits: parties - 62.7%, with peers - 37.5%, at the club – 25.2%, during vacation 18.6%<br>- Alcohol consumption on school excursions - 11.3%, when alone at home - 9.1%
The ESPAD (European School Survey Project on Alcohol and other Drugs) Report 2019 [58]	Cross-sectional study	99 647 students aged 15-16 years from 35 countries, including Romania (n=3 764)	Questionnaire<br>On use of substances and engagement in potentially addictive behaviours	- Alcohol consumption : 82.1%, increasing compared to previous studies<br>- Placing itself above the ESPAD average of 78.5%.<br>- The consumption of alcohol in the last 12 months registers the value of 74%, increasing of the values recorded in

				previous studies and still placing above the average ESPAD countries amounting to 69.4%.<br><br>-The consumption of alcoholic beverages in the last 30 days recorded higher values in Romania compared to 2015 (increasing from 47% to 51.7%). The value observed in 2019 in Romania is above the average of ESPAD countries (46.6%)

Supplementary Table 5. Included studies that address the use of tobacco and tobacco-like products.

Author, Year	Type of study	Population	Method	Main findings
Polanska K et al, 2022 [64]	Cross-sectional study	10783 aged 11-17 years, from five countries including Romania (n=3718)	Questionnaire - Global Youth Tobacco Survey	≈ 25% showed susceptibility to tobacco use in four out of five countries<br>- Correlates of susceptibility to tobacco use: exposure to environmental tobacco smoke in public venues; the smoking status of peers; the belief that smoking enhances one's comfort during celebrations; observing individuals using tobacco in mass media; limited awareness regarding the detrimental effects of passive smoking; insufficient education on anti-smoking initiatives provided by schools; family

				- Adolescents who held certain beliefs about smoking were less likely to initiate tobacco use: that smokers have fewer friends, smoking makes young people appear less attractive<br>- In all countries studied, having greater disposable income was positively associated with susceptibility to smoking (Adjusted Odds Ratio > 1.5; p < 0.01), except in Lithuania, where youth with higher disposable income were less susceptible to tobacco use (p > 0.05).<br>- In Romania, youth who believe that people who smoke have more friends were found to be more susceptible to smoking (AOR1.4; p=0.04), whereas in other countries, they tended to be less susceptible.
Albert-Lőrincz E et al 2019 [65]	Cross-sectional study	1313 aged 12-15 years	Questionnaire on smoking habits	-The most influential predictor of adolescent smoking was classmates' smoking behaviour: those with many smoking classmates were nine times more likely to smoke themselves.
Lotrean LM et al, 2021 [66]	Cross-sectional study	400 aged 18-24 years	Questionnaire on smoking habits	- Causes of smoking and using substances: become less reserved – 18.2%, feel more comfortable socialising - 8.9%, demonstrate something – 4.8%, to gain acceptance from their friends – 4.5%, being bored -20.9%
Lazea C et al, 2020 [67]	Cross-sectional study	1147 aged 13-15 years	Questionnaire on internet use, smoking, lifestyle	- 11% of the adolescents experienced palpitations and associated symptoms<br>- There is an independent correlation between palpitations and the following factors: urban residence; internet use between 8.00—12.00 pm; smoking, energy drink consumption
Tudor TE et al, 2022 [68]	Cross-sectional study	783 aged 13-14 years	Questionnaire on electronic cigarette use	- A significant majority (96.3%) of middle school students report awareness of e-cigarettes.

				- Among responders smokers were most likely to have tried e-cigarettes at least during lifetime (72.7%), followed by ex-smokers (50.8%) and non-smokers (15.4%)<br>- In the last month, 20.3% of smokers, 4.8% of ex-smokers, and 4.5% of non-smokers used e-cigarettes
Chirilă S et al, 2023 [69]	Cross-sectional study	30 aged 18-26 years	Interview	- The legal gap allowing indoor use of heated tobacco products, but not combustible cigarettes, is a contributing factor influencing young adults' choose of these products, along with perceived reduced health risks, and the product’s allure
Budin CE et al, 2022 [70]	Cross-sectional study/interventional	275 aged 10-18 years	Questionnaire and clinical measurements	- For 75% of nonsmokers, the calculated probability of being a smoker was below 20.4%, while 75% of confirmed smokers had a predicted probability exceeding 30.3%.
Budin CE et al, 2021 [71]	Cross-sectional study	275 aged 14 years	Questionnaire on tobacco use behaviours; Intervention with video materials about effects of smoking and group discussion	- Participants living in family-type houses were significantly more likely to be smokers - 36.7%, compared to those residing in the Maternal Assistance System (AMP) -11.7%.<br>- Children from the residential system were much more likely to live with smokers (78.3%) and be exposed to second-hand smoke (64.7%) compared to children in the AMP (32.9% and 31.8%, respectively).<br>- There was a significant association between the number of cigarettes smoked daily and the age of the participants (p = 0.01, rho 0.42)<br>- Among study participants, 82.3% used cigarettes and 19.4% used e-cigarettes.<br>- Most smokers obtained their cigarettes from stores - 63.2%, followed by asking friends - 19.3% or adults - 12.3% to purchase them.

Lotrean LM et al, 2013 [72]	Prospective study	1071 aged 14-15 years	Questionnaire, smoking prevention lessons	- Romanian adolescents receive insufficient anti-smoking education in schools<br>Implementing the smoking prevention program led to Romanian adolescents receiving more anti-smoking education of higher quality
Nădășan V et al, 2017 [73]	Prospective study	1369 aged 14-15 years	Questionnaire, smoking prevention lessons with ASPIRA program	- The intervention program reduced the risk of smoking initiation by 35% among never-smoker students within 6 months (OR = 0.65, 95%CI: 0.44–0.97).<br>- Students who participated in at least 75% of the ASPIRA program showed the greatest reduction in smoking initiation<br>- The intervention did not have a significant impact on current tobacco use
Nădășan V et al, 2019 [74]	Cross-sectional study	675 aged between 14-15 years	Questionnaire	- 68.3% of the responders were strongly influenced by the program<br>- Students were more likely to receive high program exposure if they met these criteria: their father had a lower level of education, their brother was a nonsmoker, they had never tried e-cigarettes, they strongly valued the health of others
Banzer R et al, 2017 [75]	Cross-sectional study	12,328 aged 13-17 from 11 countries. (Romania N=1140)	Questionnaire - "saving and empowering young lives in Europe" (SEYLE) project	- Adolescent smoking remains a serious public health concern, as it significantly increases the risk of mental health problems in young people<br>- Family instability and parental behaviours can contribute to adolescent smoking
The ESPAD (European School Survey Project on Alcohol and other Drugs) Report 2019 [58]	Cross-sectional study	99 647 students aged 15-16 years from 35 countries, including Romania (n=3 764)	Questionnaire on use of substances and engagement in potentially addictive behaviours	- For all reference periods – lifetime, in the last month and daily consumption in the last month, tobacco consumption among teenagers in Romania is maintained at similar values to those observed in the previous study.

				- Lifetime tobacco use among adolescents continues the downward trend started since 2003, decreasing successively from 64%, to 54%, to 52%, respectively to 51.7% and reaching in 2019, to 49.5%.<br>- The consumption of cigarettes in the last 30 days maintains a level similar to that observed in 2011, reaching 31.2%.<br>- The daily consumption of cigarettes in the last 30 days returns to the value recorded in 2011, respectively 18.4%.

Supplementary Table 6. Included studies that address the mental health and well-being situation (both before and after the COVID-19 pandemic) and online activities.

Author, Year	Type of study	Population	Method	Main findings
Sârbu EA et al, 2022 [77]	Cross-sectional study	2690 aged between 15-16 years	Questionnaire from Youth in Europe Study	- Link between exposure to adversity and mental health problems, with increased frequency, intensity, and earlier onset of negative events leading to more severe depression and anxiety in adolescents<br>- More frequent, recent, and intense negative experiences in life were strongly linked to increased depression symptoms in adolescents (35.8%)
Popescu CA et al, 2023 [78]	Cross-sectional study	1061 aged between 18-24 years	Questionnaire to	- Depression scores decreased significantly from 2018 to 2022 (mean scores: 13.81 vs. 11.56), with a high level of statistical confidence ($p < 0.001$)

			explore the impact of stress, substance use, the pandemic, and online education on mental health.	- Students in 2018 reported significantly higher levels of stress across various areas of their lives<br><br>- Key factors that increase the likelihood of academic stress and depression among students: being female, experiencing financial difficulties, and being in pre-clinical years of study<br>- Losing a family member to COVID-19 was associated with a significant increase in both depression scores (measured by the BDI) and current anxiety levels among students
Dumitrache L et al, 2021 [79]	Cross-sectional study	722 aged between 18-24 years	Questionnaire -<br><br>The Romanian version of DASS-21<br><br>- The Boredom Proneness Scale–Short Form (BPS-SR)<br><br>- The Missing Daily Social Interactions' Scale (DSIMS)<br><br>- Pandemic-related questions.	- Higher stress levels were linked to boredom, missing social interactions, more phone calls, and increased interest in pandemic news<br>- Students who are easily bored, miss social interaction, spend excessive time on the phone, and closely follow pandemic news tend to report higher levels of perceived stress

Pop LM et al, 2022 [80]	Cross-sectional study	427 aged 18-24 years	Questionnaire<br><br>on image of their own body,self-esteem, social media	- Nearly half - 49% of students use social networking sites (SNS) for socializing, followed by entertainment - 31.1% and academic tasks - 19.9%. On average, they spend 3.38 hours (± 0.80) a day on SNSs.<br><br>- Slightly less than half of the students - 47.5% compared themselves to others on social media.<br><br>- Snapchat use was linked to higher self-esteem, while TikTok use was associated with lower weight status<br>- Over 75% of participants reported exercising for weight loss or weight control
Iovu MB et al, 2020 [81]	Cross-sectional study	708 aged 13-35 years	Questionnaire - some measures of Facebook addiction, depression, and family satisfaction: Bergen Facebook Addiction Scale (BFAS), Beck's Depression Inventory (BDI), Family Satisfaction by Adjectives Scale (FSAS)	- Increased Facebook use is associated with decreased satisfaction with family life<br>- Increased Facebook use is associated with more pronounced symptoms of depression<br><br>- More intense Facebook use is associated with a higher risk of an addictive relationship with the platform.
Maftei A et al, 2023 [82]	Cross-sectional study	461 aged 18-25 years		- Links between all types of perfectionism and symptoms of internet addiction

			Questionnaire on subjective happiness, perfectionism, substance use, internet addiction	- Narcissistic perfectionism and self-critical along with addictive behaviours, were linked to lower levels of happiness<br>- The negative impact of perfectionism on happiness is primarily explained by unhealthy behaviours adopted as coping mechanisms<br>- Chain reaction: high levels of perfectionism led to increased health-risk behaviours, which in turn were associated with lower levels of happiness
Drosopoulou G et al, 2023 [83]	Cross-sectional study	8952 aged 14-17,9 years from 5 countries, Romania (n=1830)	Questionnaire - Youth Self Report Questionnaire -<br><br>Skills to handle the everyday demands of life<br><br>Demographic Questionnaire,	- Iceland had the highest rate of internalizing problems -11.1%, while Greece -7.6% and Romania - 2.4% had the lowest<br>- Internalizing problems were linked with poorer school performance.<br>- Higher parental education was linked to a reduced likelihood of internalizing problems<br>- Factors like gender, age, and whether a parent had a job were linked to how likely someone was to experience internalizing problems
Maftei A et al, 2022 [84]	Cross-sectional study	155 aged 10-13 years	Questionnaire on<br><br>The perceptions of the students of COVID-19 pandemia and online learning	- A significant portion -41.3% of children and teens linked masks to COVID-19. Sadness was the most common feeling - 45.2%, fear -17.4%<br>- Participants reported moderate levels of pandemic-related hardship, mainly due to the switch to online classes<br>- Children who felt the pandemic had a harmful effect on them were more likely to perceive negative consequences for their families as well

				- The majority of participants were unhappy with online classes, particularly citing a lack of meaningful interaction as the main reason<br>- Participants primarily relied on positive thinking and family connections to cope with the pandemic, suggesting they saw similar strategies used by those around them<br>>50% did not see any positive aspects of the COVID-19 pandemic. However, 40% viewed increased family time as the main benefit arising from the health crisis
Maftei A et al, 2020 [85]	Cross-sectional study	73 aged 5-18 years and 73 parents aged 29-47 years	Questionnaire on The Parenting Styles and Dimensions Questionnaire (PSDQ), Interviews with children individually and parents	- The majority of children believe that following family rules is what makes their parents happiest<br>- Children's happiness primarily links to closed relations with family and friends.<br>- Strong connections between children's happiness, parents' happiness, and certain aspects of both permissive and authoritarian parenting styles
Iuga IA et al, 2023 [86]	Cross-sectional study	602 aged 8-16 years	Questionnaire on The School Burnout Inventory, The Emotion (SBI) Regulation Index for Children and Adolescents (ERICA), The Security Scale	- Secure attachment was found to offer protection against burnout. Participants with strong attachment bonds were less likely to experience exhaustion, cynicism, and feelings of inadequacy<br>- Greater emotional control was significantly associated with lower levels of exhaustion, cynicism, and inadequacy – key symptoms of burnout<br>- Greater emotional awareness significantly reduced the likelihood of experiencing emotional exhaustion, cynicism, and feelings of inadequacy<br>- Secure attachment was significantly linked to better emotional control and a stronger sense of emotional awareness

Jankowiak B et al, 2020 [88]	Cross-sectional study	993 aged 13-16 years from from 6 countries, Romania (n=190)	Questionnaire - Student Social Support Scale (CASSS), School Social Climate (CECSCE)	- Young victims of dating violence or those fearing their partner had lower overall social support<br>- More social support at school was linked to a decreased risk of dating violence (physical or sexual) among young people<br>- A more positive school climate was linked to a decreased risk of fear among young people<br>- Adolescents in Europe experiencing dating violence reported lower school social support and climate
Stoian CE, 2022 [90]	Cross-sectional study	383 aged 18-22 years	Questionnaire on perception about online education during the Pandemic	- Students valued teacher support with technology, online learning platforms, better teacher communication, and peer collaboration
Ghergeș V et al, 2021 [91]	Cross-sectional study	604 aged 18-22 years	Questionnaire on<br><br>online leaning versus face-to- face learning	- Preferences for studying: 27.2% face-to-face learning, 13.9% online learning<br>- The top three e-learning benefits, according to adolescents, are time efficiency -15.7%, convenience - 14.7%, and accessibility -11.6%<br>- Students reliant solely on e-learning were nearly twice as likely to express dissatisfaction -16.6% compared to those with other options - 8.9%.<br>- Surprisingly, students with access to face-to-face learning perceived e-learning as being more engaging
Hinoveanu D et al, 2023 [92]	Cross-sectional study	85 aged 16-19 years	Questionnaire on mental health, substance use, anxiety and academic results	-Significant improvements in several areas between the academic years 2020-2021 and 2022-2023: COVID-19 vaccination rates doubled (18.2% to 39.0%, $p = 0.033$), and students felt much better

				supported in mental health during remote learning (7.1% to 20.0%, p = 0.044).<br><br>- Students' anxiety about returning to in-person learning significantly decreased (p = 0.048)<br>- Standard health surveys showed positive trends in both physical and mental health (p = 0.046, p = 0.019, respectively). Anxiety symptoms also significantly decreased (p = 0.038). Also, mental well-being improved (p = 0.039)
Lotrean LM et al, 2023 [144]	Cross-sectional study	306 aged 22-25 years	Questionnaire on Attitudes and Intentions Regarding Digital Health training	- Medical students need a formal digital health training program during their education.<br>-Students are eager to use digital health tools, supporting further digitalization within healthcare. Proper training for medical professionals will ensure maximum benefit for patients and the entire system

Supplementary Table 7. Included studies that address sexual education and reproductive health.

Author, Year	Type of study	Population	Method	Main findings
Grad AI et al, 2018 [99]	Cross-sectional study	3872 aged 18-25 years	Questionnaire regarding sexual behaviour, attitudes, and knowledge about STIs	- Age debut of sexual activity: 14-18 years for 53.7%; 2.3% under 14<br>- 30% of the male and female participants had more than one sexual partner in the previous year and significant portion of participants in this study (≈25%), especially those who began sexual activity earlier, did not use contraception during their first sexual encounter

				- The data shows a concerning number of students are engaging in behaviours that could increase their risk of unintended pregnancy and sexually transmitted infections (STIs)
Molnar A et al, 2020 [100]	Observational study	939 aged 15-19 years	The sexually transmitted infections (STI) surveillance system was used to collect the data	Between 2005 and 2017, reported cases of syphilis (773) and gonorrhoea (166) decreased in number. Most syphilis diagnoses resulted from active detection efforts<br>Passive detection primarily identified Gonorrhoea cases. Syphilis and gonorrhoea affected similar age groups. Syphilis occurred more frequently in females, and in rural areas, while Gonorrhoea was more common in males and in urban areas.
Voizădan TS et al, 2022 [105]	Cross-sectional study	1363 aged 17-24 years	Questionnaire on level of information about HPV infection and vaccination	- Respondents relied on different primary sources for STI information, influenced by their individual profiles and professions<br>- The source of STI knowledge varied: medical students sought guidance from doctors (53.0%), doctors used specialized materials (61.6%), and other respondents primarily relied on the Internet<br>- Despite most respondents believing doctors should be the source of HPV information and vaccination advice, surprisingly few actually consult their general practitioner about it
Iova CF et al, 2023 [106]	Cross-sectional study	690 aged 18-19 years	Questionnaire on knowledge about HPV , attitudes toward vaccination	- Pro-vaccinated girls demonstrated greater knowledge of HPV vaccination and were more open to receiving it.<br>- Despite awareness of HPV transmission and the value of vaccination, the GNV (non-vaccinated group) group lacked knowledge about HPV vaccine effectiveness, leading to their non-vaccination

				- Adolescents in the GPV group embrace HPV vaccination, with participants either already vaccinated or expressing interest in receiving it
Gismondi M et al, 2021[107]	Cross-sectional study	736 aged 18-44<br><br>From 56 countries, including Romania<br><br>(n not specified)	Questionnaire on - on knowledge about HPV and cervical cancer	- HPV was the most cited risk factor (28.8%), with females demonstrating greater awareness of the risk compared to males ($p < 0.05$)<br>- Surprisingly, senior students were less likely than junior students to identify HPV as a cervical cancer risk factor ($p < 0.05$)<br>- Asian students were significantly less likely to identify HPV as a cervical cancer risk factor compared to Western and Eastern European students
Anton-Păduraru DT et al, 2020 [110]	Prospective study	534 aged 14-18 years	Questionnaire, Health education lessons	- While girls rely on family for STD information, they lack knowledge about specific STDs beyond HIV/AIDS and how to prevent them<br>- Condoms were cited as the primary method of protection against pregnancy, rather than against sexually transmitted diseases (STDs)
Van der Starre T, 2017 [113]	Cross-sectional study	Girls under 20	Data were collected by means of the United Nations Statistics Division Demographic Yearbook database and Health for All European database	Although the overall percentage of live births from mothers under 20 declined from 13.11% in 2000 to 9.51% in 2014, there were temporary increases in the number of these births between 2002-2004 and 2007-2008
Radu CM et al 2022 [114]	Cross-sectional study	100 aged under 18 years and 100 aged over 18 years	Questionnaire - The Short Assessment of Health Literacy (SAHL)	– The group of adolescents was aged between 13-18 years. 28% gave birth between 13-15 years.<br>- The observed variation in the number of medical examinations suggests that the groups investigated may experience differing levels of prenatal care during pregnancy.

				-Low health literacy in group of adolescents, correlates with a significantly higher percentage of low-birth-weight children<br>Control group had higher gestational age (38.4 vs 36.9 weeks)
Gambadauro P et al, 2018 [115]	Cross-sectional study	12395, median age 15 from 11 European countries including Romania (n not specified)	Questionnaire "Saving and Empowering Young Lives in Europe" (SEYLE) project.	– Age of first sexual contact was frequently found to be above 15 years – 38%, than under 15 – 13.2%, and boys – 31.3% rather than girls16.9%<br>-Students with depression, anxiety, suicidal thoughts, self-harm, or suicide attempts were more likely to engage in risky sexual behaviours<br>- The connection between mental health issues and risky sexual behaviour was especially evident in younger students (15 or younger) and in females who did not exhibit other risky behaviours<br>- This study highlights how mental health problems like depression and anxiety can significantly increase the risk of complications with reproductive health.
Faludi C et al, 2019 [116]	Cross-sectional study	1359 people aged 18–30 years	Questionnaire, Interview on sexual behaviour and health risks	- Open communication about sex within the family environment was positively associated with a healthy transition into sexual activity for both females and males<br>- An absence of familial sex education is associated with a lower likelihood of experiencing a healthy sexual debut. This finding applies to both women and men<br>- Qualitative findings indicated that young people typically initiated SRH conversations with a same-gender parent. These discussions often took place following milestones in the young person's sexual

				development, such as first menstruation or sexual debut.
Pența MA et al, 2020 [126]	Cross-sectional study	401 aged 18-26 years	Questionnaire, interview on intentions to vaccinate against HPV and the seasonal flu	– The models were able to explain 51% of why people might choose to get the HPV vaccine and 60% of why they might choose to get the seasonal flu vaccine<br>- The most important factor influencing the decision to vaccinate, in both cases, was the fear of regretting inaction if an infection occurred without vaccination. This surpassed even traditional risk-related beliefs<br>- People were also more likely to get vaccinated if they believed the vaccine was effective and safe, felt personally at risk of the disease, and hadn't refused vaccines in the past<br>- This study reveals how much young adults know about vaccines and the ways they find vaccine-related information

Supplementary Table 8. Included studies that address access to health services and preventive medicine in terms of health promotion and disease prevention.

Author, Year	Type of study	Population	Method	Main findings
Hammami N et al, 2022 [129]	Cross-sectional study	228979 aged 11 to 15 years from 45 countries including Romania	Questionnaire Health Behaviours in School-aged Children (HBSC) survey	- The study revealed three distinct profiles of health complaints: one with no reported complaints, one focused on psychological symptoms, and one with a combination of physical and psychological symptoms

				- Among the health complaint classes, the class with both physical and psychological complaints had the lowest socioeconomic status<br>- Health initiatives for adolescents should prioritize the unique needs of those from low-income households, as they tend to experience more health concerns
Turaiche M et al, 2022 [125]	retrospective cohort analysis study	104 under 10 and 32 between 10-18	- The study used clinical and laboratory records from patients treated for infectious diseases at "Victor Babes" Hospital in Timisoara	-Roma patients, despite being a Romanian minority, were strikingly overrepresented in this study (over 40%)<br>- Children under 10 years old were more likely to contract the infection from within their family than adolescents. This suggests that transmission dynamics may differ between these two age groups.<br>- The study found that older age, poor nutrition, Roma ethnicity, anaemia, and high procalcitonin levels were all significant, independent risk factors for pneumonia
Pacurari, N et al, 2021 [124]	Prospective study		To gather diverse perspectives, the study conducted focus groups with healthcare providers and patient representatives from multiple paediatric oncology centres across Romania	- For healthcare professionals, the emotional demands of the job, limited work-life balance, and insufficient staffing were major obstacles to successfully implementing paediatric palliative care.<br>- Financial limitations and the stigma surrounding palliative care and oncology were cited as the primary reasons for understaffing<br>- Political instability was also identified as a significant barrier to implementing palliative care
Durach F et al, 2022 [127]	Literature review	-	Literature search on vaccine intentions, social media, immune system, hesitancy reasons	- Vaccination decisions differ for adolescents and their parents, and these decisions become more complex for children with neurological disorders.

				Parents may fear that vaccination could worsen their child's condition, and physicians may lack specialized knowledge.<br>The current pandemic and spread of misinformation on social media present significant challenges to vaccine acceptance, but these platforms can also be used to combat misinformation and promote the benefits of vaccination
Rogobete SE et al, 2021 [129]	Cross sectional	987 aged 14-18 years	Questionnaire on attitudes regarding abortion, euthanasia and human rights	- Church attendance and prayer influence attitudes towards euthanasia and abortion similarly – those who attend church and pray frequently are the least accepting of these practices<br>However, church attendance and prayer have contrasting effects on views about socio-economic human rights. Regular church attendance (extrinsic religiosity) correlates with less support for such rights, while frequent prayer (intrinsic religiosity) correlates with greater support
Marin DC et al, 2020 [130]	Literature Review	-	Literature search on implementation of the health education programs	- There were two national projects on health education released, one is optional for schools and the other one has the framework, but still waiting for the guidelines for implementation<br>Within Romania, there is limited quantitative research and government analysis evaluating the impact of health education initiatives
